# *In Silico* Identification of Carboxylate Clamp Type Tetratricopeptide Repeat Proteins in *Arabidopsis* and Rice As Putative Co-Chaperones of Hsp90/Hsp70

**DOI:** 10.1371/journal.pone.0012761

**Published:** 2010-09-15

**Authors:** Bishun D. Prasad, Shilpi Goel, Priti Krishna

**Affiliations:** Department of Biology, The University of Western Ontario, London, Ontario, Canada; University of Heidelberg, Germany

## Abstract

The essential eukaryotic molecular chaperone Hsp90 operates with the help of different co-chaperones, which regulate its ATPase activity and serve as adaptors to recruit client proteins and other molecular chaperones, such as Hsp70, to the Hsp90 complex. Several Hsp90 and Hsp70 co-chaperones contain the tetratricopeptide repeat (TPR) domain, which interacts with the highly conserved EEVD motif at the C-terminal ends of Hsp90 and Hsp70. The acidic side chains in EEVD interact with a subset of basic residues in the TPR binding pocket called a ‘carboxylate clamp’. Since the carboxylate clamp residues are conserved in the TPR domains of known Hsp90/Hsp70 co-chaperones, we carried out an *in silico* search for TPR proteins in *Arabidopsis* and rice comprising of at least one three-motif TPR domain with conserved amino acid residues required for Hsp90/Hsp70 binding. This approach identified in *Arabidopsis* a total of 36 carboxylate clamp (CC)-TPR proteins, including 24 novel proteins, with potential to interact with Hsp90/Hsp70. The newly identified CC-TPR proteins in *Arabidopsis* and rice contain additional protein domains such as ankyrin, SET, octicosapeptide/Phox/Bem1p (Phox/PB1), DnaJ-like, thioredoxin, FBD and F-box, and protein kinase and U-box, indicating varied functions for these proteins. To provide proof-of-concept of the newly identified CC-TPR proteins for interaction with Hsp90, we demonstrated interaction of AtTPR1 and AtTPR2 with AtHsp90 in yeast two-hybrid and *in vitro* pull down assays. These findings indicate that the *in silico* approach used here successfully identified in a genome-wide context CC-TPR proteins with potential to interact with Hsp90/Hsp70, and further suggest that the Hsp90/Hsp70 system relies on TPR co-chaperones more than it was previously realized.

## Introduction

The eukaryotic Hsp90 performs key roles in signal transduction by regulating maturation, localization, stability and protein interactions of a large number of signaling proteins [1], [2]. Due to its role in chaperoning oncogenic protein kinases, Hsp90 has recently been surfaced as a drug target for inhibiting cancer progression in humans [3]. Additional roles of Hsp90 lie in chromatin remodeling, epigenetic regulation, and morphological evolution [2]. Eukaryotic Hsp90 functions as a homodimer with each monomer comprising of an N-terminal ATP-binding domain that is responsible for the ATPase activity of Hsp90, a middle domain that harbors the client protein binding site and can also regulate the ATPase activity of the N-terminal domain, and a C-terminal dimerization domain [4]. Hsp90-specific inhibitors such as geldanamycin (GA) bind to the N-terminal domain of Hsp90 and inhibit its *in vivo* functions [3]. The C-terminus of cytosolic Hsp90 contains a conserved pentapeptide MEEVD, which is responsible for binding with the tetratricopeptide repeat (TPR) domain present in several co-chaperones of Hsp90 [1], [5].

Hsp90 has an ATP-driven chaperone cycle [2]. The open V-shaped conformation of dimeric Hsp90 allows for loading of the client protein. Following binding of ATP, a conformational change in the middle domain leads to a closed conformation in which the monomers are twisted around each other and the two N-terminal domains are dimerized. With the coordinated assistance of Hsp70 and a range of co-chaperones in dynamic protein heterocomplexes, the final maturation of the client protein takes place. Upon ATP hydrolysis, the client protein and the heterocomplex dissociate and Hsp90 enters a new chaperone cycle [4].

The various co-chaperones assist Hsp90 by influencing its ATPase activity and by linking it to other proteins and providing some measure of specificity to the client protein-Hsp90 interaction [6]. On a structural basis the Hsp90 co-chaperones can be broadly divided into two categories: TPR domain proteins and non-TPR proteins. The TPR domain is a protein-protein interaction domain, comprised of a loosely conserved 34 amino acid sequence that is repeated several times [7]. Several Hsp90 co-chaperones, such as Hsp70-Hsp90 organizing protein (Hop), high molecular weight immunophilins [cyclophilin 40 (Cyp40) and FK506-binding proteins 51 and 52 (FKBP51 and FKBP52)], protein phosphatase 5 (PP5) and the carboxyl terminus of Hsc70 interacting protein (CHIP) contain a three-motif TPR domain with conserved carboxylate clamp residues [5]. Within a TPR motif, eight amino acids at positions 4 (W/L/F), 7 (L/I/M), 8 (G/A/S), 11 (Y/L/F), 20 (A/S/E), 24 (F/Y/L), 27 (A/S/L), and 32 (P/K/E) have a higher frequency of conservation and these are important in maintaining the α-helical structures within a motif. Clustering of several α-helices within a tandem array of TPR motifs generates an amphipathic channel with a large amount of surface area, which allows the TPR domain to recognize its target protein [7].

A good understanding of the interaction of the TPR domain with molecular chaperones Hsp90 and Hsp70 came from cocrystalization study of TPR domains in Hop with pentapeptide MEEVD and octapeptide GPTIEEVD, which correspond to the C-termini of Hsp90 and Hsp70, respectively [8]. The side chains of several basic amino acids in the TPR groove were seen to establish interactions with the acidic side chains of the EEVD motif. These basic amino acids are referred to as ‘carboxylate clamp’ residues. With reference to the positions of the clamp residues in a three-motif TPR domain, the consensus is Lys_5_ and Asn_9_; Asn_6_; Lys_2_ and Arg_6_. These amino acids are conserved and functionally important in most three-motif TPR containing co-chaperones of Hsp90 and Hsp70. Additional contacts between EEVD and the TPR groove involve hydrophobic interactions [9].

Hsp90 has been studied widely in animal and yeast model systems, but little is known by comparison about plant Hsp90. The Hsp90 family in the model plant *Arabidopsis thaliana* comprises of seven members: four closely related isoforms are cytosolic (AtHsp90-1 to AtHsp90-4), one is chloroplastic (AtHsp90-5), one mitochondrial (AtHsp90-6), and one is localized to the endoplasmic reticulum (ER) (AtHsp90-7) [10]. The occurrence of multiple Hsp90 isoforms that display both developmental and stress-responsive gene expression, suggests a range of specific functions for these proteins. The recent demonstration of the involvement of Hsp90 and co-chaperones RAR1 (required for M1a12 resistance) and SGT1 (suppressor of G2 allele of *skp1*), in plant disease resistance [11], [12] has provided impetus for further investigation of the roles of Hsp90 in plant signaling pathways. Apart from RAR1 and SGT1, Hsp70 [13], high molecular weight immunophilins [14], Hop-like protein [15], PP5 [16], and p23-like proteins [17] have been found to associate with plant Hsp90. Plant orthologs of the previously characterized Hsp90/Hsp70 co-chaperones with TPR domains include Hop [15], PP5 [16], immunophilins like Cyp40 (SQUINT) [18], Rotamase AtFKBP62 (AtROF1) and AtFKBP65 (AtROF2) [19], PASTICCINO1 (AtPAS1) [20], AtFKBP42 (AtTWD1) [21], [22], Translocon of the outer envelope of chloroplast (TOC64) [23] and AtCHIP [24]. Given the conservation and preponderance of the TPR domain in established and potential co-chaperones of the plant Hsp90/Hsp70 system, we set out to uncover all carboxylate clamp (CC) type TPR proteins (CC-TPR proteins) in *Arabidopsis* using an *in silico* approach and following the residues conserved for interaction with the EEVD motif of cytosolic Hsp90/Hsp70. Here we report the identification of 24 new CC-TPR proteins in *Arabidopsis*, and similar as well as distinct CC-TPR proteins in rice (*Oryza sativa*), as putative Hsp90/Hsp70 interactors. Some of the newly uncovered proteins contain additional protein domains such as ankyrin, SET, octicosapeptide/Phox/Bem1p (Phox/PB1), DnaJ, thioredoxin, FBD and F-box, and protein kinase and U-box, indicating novel functions that may add new dimensions to the Hsp90/Hsp70 chaperone complex in plants.

## Methods

### Identification of CC-TPR proteins in *Arabidopsis*

The term ‘TPR’ submitted as query at the InterPro home page: http://www.ebi.ac.uk/interpro/ [25] retrieved 31 entries of which IPR013026 was found to best conform to TPR structure and function. The ‘Taxonomic coverage’ of IPR013026 revealed 235 TPR proteins in the *Arabidopsis* proteome (taxon ID 3702). The sequences of all proteins and the *Arabidopsis* Genome Initiative Identifier (AGI ID) were recorded and confirmed against the database of National Centre for Biotechnology Information (NCBI) and each sequence was analyzed for the presence of TPR domain using InterProScan (http://www.ebi.ac.uk/InterProScan/) [26]. Since the known co-chaperones that interact with MEEVD of Hsp90 consist of three-motif TPR domain, proteins with one or more TPR domains comprising of three motifs were short-listed. Subsequently, these motifs were analyzed for the presence of the conserved residues (K_5_N_9_-N_6_-K_2_R_6_). A second round of search was carried out where protein sequences with one or two TPR motifs were manually mapped for the presence of the second and/or third motif and the conserved residues (K_5_N_9_-N_6_-K_2_R_6_). Following selection of the CC-TPR proteins, these proteins were searched for additional known domains and localization signal sequences. Multiple sequence alignment for each of the three motifs of the CC-TPR proteins identified in *Arabidopsis* (above-described search) and human Hop TPR2a as the reference sequence was used to build a statistical model of the corresponding motif using the software package HMMER 3.0 (http://hmmer.org/). Each of these three models was queried on the basis of HMM profile against TAIR9_pep_20090619 (*Arabidopsis* annotation from TAIR) and Rice Genome Annotation Project (ftp://ftp.plantbiology.msu.edu/pub/data/Eukaryotic_Projects/O_sativa/annotation_dbs/pseudomolecules/version_6.0). The HMMER-selected proteins (E-value <10) were scanned for conserved residues (K_5_N_9_-N_6_-K_2_R_6_), and BLAST and manual editing were used to remove redundancy.

### Phylogenetic tree construction

Full-length sequences of CC-TPR proteins of *Arabidopsis* and rice were aligned using Clustal X 2.0.10. The phylogenetic tree was derived from the sequence comparisons using the neighbor-joining method in Clustal X.

### Protein localization prediction

The electronic Fluorescent Pictograph (eFP) Browser [27] from the Bio-Array Resource (BAR) (http://bbc.botany.utoronto.ca/) was used to predict the subcellular localization (SL) of the CC-TPR proteins. The information retrieved was based on computational prediction and/or experimental documentation compiled in SUBA; the *Arabidopsis* SUBcellular localization database [28]. If experimental documentation for SL was available for a protein, it was considered as significant. However, for proteins lacking experimental documentation we inferred data available from several prediction algorithms such as iPSORT (http://hc.ims.u-tokyo.ac.jp/iPSORT/), LOCtree (http://cubic.bioc.columbia.edu/cgi-bin/var/nair/loctree/query), MitoPred (http://bioapps.rit.albany.edu/MITOPRED/), MitoProt II (http://ihg2.helmholtz-muenchen.de/ihg/mitoprot.html), MultiLoc (http://www-bs.informatik.uni-tuebingen.de/Services/MultiLoc/), PeroxP, Predotar (http://urgi.versailles.inra.fr/predotar/predotar.html), SubLoc (http://www.bioinfo.tsinghua.edu.cn/SubLoc/), Target P (http://www.cbs.dtu.dk/services/TargetP/) and WOLFPSORT (http://wolfpsort.org/).This data was cross-checked with the actual eFP Browser data that was generated using heuristic prediction algorithms [27].

### Expression analyses using AtGenExpress Visualization Tool

Affymetrix microarray data provided by Weigel World (http://www.weigelworld.org/) was accessed using AtGenExpress Visualization Tool (AVT) (http://www.weigelworld.org/resources/microarray/AtGenExpress/) [29], [30] to analyze the developmental expression of the newly identified *CC-TPR* genes by inputting their AGI IDs on the homepage. Absolute expression values were retrieved and used to develop the figure.

### Expression analysis using MPSS database

The MPSS database (http://mpss.udel.edu/rice/) was searched (opting 17-nt signature sequences) using the locus ID given in the Rice Genome Annotation Project database to generate the expression profiles of rice CC-TPR proteins in different tissues and stress.

### Electronic Northern Analysis

The expression profiles of *Hsp90* and *CC-TPR* genes were examined against the AtGenExpress extended tissue and abiotic stress data sets using the Expression Browser tool available at the Botany Array Resource (BAR) (http://bbc.botany.utoronto.ca/affydb/cgi-bin/affy_db_exprss_browser_in.cgi) [31]. The output option was selected as average of replicate treatments relative to the average of appropriate control. The output files were formatted into heatmaps using the Data MetaFormatter tool hosted at the BAR website by selecting ‘View Graphical Representation of Log Transformed Cluster Data’. The colour scale indicates the log_2_-level of expression above or below the median. Strong red indicates more than four-fold above the median, while dark blue indicates four-fold below the median.

### Expression Angler

The Expression Angler tool at BAR identifies genes that are co-expressed with a gene of interest [31]. Using the cut-off r-value between 0.75–1.00, 156 genes were found against the AtGenExpress stress series dataset to be co-expressed with *AtHsp90-1* (*AT5G52640*), a bona fide heat stress (HS)-induced gene. This list of genes was checked against the *Arabidopsis* list of 36 CC-TPR proteins to identify those that are co-expressed with *AtHsp90-1* under HS.

### Plant growth and treatments

*Arabidopsis* seeds were surface sterilized and plated on 1X Murashige and Skoog (MS) medium (Sigma) supplemented with B5 vitamins, 1% (w/v) agar and 1% sucrose. The plates were kept for 3 days in the dark at 4°C to encourage synchronized germination and then transferred to a growth chamber maintained at 22°C with a 16/8 h photoperiod (80 µmol m-2 s-1). For HS treatment, 10 day-old seedlings were exposed to 38°C for 1 h and 3 h in an incubator, following which the plant tissue above the medium was collected and quick-frozen for RNA isolation. For treatment with brassinosteroid (BR), 21 day-old seedlings grown on MS medium were submerged in water containing either 1 µM 24-epibrassinolide (EBR) or 0.01% ethanol (solvent for EBR) for 3 h and 12 h. After the treatment, the plant material was quick-frozen.

### RNA extraction and Quantitative real-time RT-PCR

Total RNA was prepared using SV Total RNA Isolation System (Promega). First strand cDNA was synthesized from 1 µg of total RNA using QuantiTect Reverse Transcription Kit (Qiagen) and used as a template for quantitative RT-PCR (qRT-PCR). qRT-PCR was performed in 200 µl tubes with a Rotor-Gene RG-3000 real-time thermal cycling system (Corbett Research) using SYBR green to monitor double-strand DNA synthesis. Three independent biological samples were used with gene-specific primers (Table S1). Data were analyzed using Rotor-Gene 6.0.16 software (Corbett Research). Values were normalized using ubiquitin as the internal reference, and fold change in the expression level was calculated [32].

### Yeast two-hybrid assay

To determine interaction of the two newly identified proteins, AtTPR1 and AtTPR2, with AtHsp90-2 by the yeast two-hybrid approach, *AtHsp90-2* and *AtTPR1/AtTPR2* coding sequences were cloned into bait and prey vector, respectively. *AtHsp90-2* was amplified by PCR using the *Arabidopsis* Biological Resource Center (ABRC) cDNA clone C105057 and primers attB1-Hsp90-2F 5′- GGGG ACA AGT TTG TAC AAA AAA GCA GGC TCC ATG GCG GAC GCT GAA ACC TTT GCT TTC-3′ and attB2-Hsp90-2R 5′- GGG GAC CAC TTT GTA CAA GAA AGC TGG GTC GTC GAC TTC CTC CAT CTT GCT ACC TTC -3′. The PCR product was cloned into pDONR221 by *in vitro* BP recombination (Invitrogen) to generate pDONRHsp90-2, which was used in LR reaction with pDestDB (bait vector) to generate pDBHsp90-2. *AtTPR1* and *AtTPR2* cDNAs were synthesized from total RNA from *Arabidopsis* leaf using gene-specific gateway primers (attB1-TPR1F 5′- GGGG ACA AGT TTG TAC AAA AAA GCA GGC TCC ATG GTA CTG ATC GAA TCA AGT GAG AG-3′ and attB2-TPR1R 5′- GGGG GAC CAC TTT GTA CAA GAA AGC TGG GTC TA TGG CTC TTC CAC TAA ACC CG-3′; attB1-TPR2F 5′-GGGG ACA AGT TTG TAC AAA AAA GCA GGC TCC ATG GCG CTA TGG ATG GAC GCT GG-3′ and attB2-TPR2R 5′-GGG GAC CAC TTT GTA CAA GAA AGC TGG GTC GTT TGG TGG AGT CCA TTT TCC AGC G-3′). *AtTPR1* and *AtTPR2* PCR products were cloned, as described for AtHsp90-2, into pDestAD (prey vector) to generate pADTPR1 and pADTPR2. Following sequence verification, pDBHsp90-2 and pADTPR1/pADTPR2 were transformed into Y8930 and Y8800 yeast strains, respectively. Transformants were selected on synthetic complete (SC) media lacking either leucine (bait vector) or tryptophan (prey vector). Bait and prey transformants were then mated and selected on SC media lacking leucine and tryptophan. The interaction of pDBHsp90-2 with pADTPR1/pADTPR2 was assayed based on the ability of cells to grow on SC-Leu-Trp-His plus 3 mM 3-aminotriazole (3AT).

### *In vitro* protein-binding assay

Plasmid expressing AtHsp90-2 with a cleavable N-terminal glutathione-S-transferase (GST) tag in pGEX-6p-1 (Hsp90-GST) was kindly provided by Dr. David Hubert, University of North Carolina. Plasmid expressing Hsp90ΔMEEVD-GST was generated by PCR amplification and cloning of the PCR product into pGEX-6p-1 (GE Healthcare). The chitin-binding domain (CBD) affinity tag was fused to the C-terminus of TPR1 by cloning TPR1 cDNA in frame into pTYB2 (New England Biolabs) to prepare TPR1-CBD. Similarly, the GST tag was fused to the N-terminus of TPR2 by cloning TPR2 cDNA in frame into pGEX-6p-1 to prepare TPR2-GST fusion protein.

Hsp90-GST and Hsp90ΔMEEVD-GST were expressed in BL21 cells. Expressed proteins were purified on Glutathione-Sepharose 4B beads as per the protocol provided by GE Healthcare. The beads were washed once with PBS buffer containing 500 mM NaCl, five times with PBS buffer, and once with cleavage buffer (50 mM Tris, pH 7.0, 150 mM NaCl, 1 mM EDTA and 1 mM DTT). The beads were incubated at 4°C overnight with cleavage buffer containing PreScission Protease to cleave the GST tag. The cleaved protein was eluted with the cleavage buffer. Fresh Glutathione-Sepharose 4B beads were added to the eluate (cleaved Hsp90 and Hsp90ΔMEEVD) to remove the PreScission Protease. Purified Hsp90 and Hsp90ΔMEEVD were concentrated, dialyzed against 10 mM HEPES, pH 7.5, and 50 mM NaCl, and stored at −80°C until further use.

TPR1-CBD and TPR2-GST proteins were produced in *E. coli* strains ER2566 and BL21 cells and immobilized on chitin beads and Glutathione-Sepharose 4B beads, respectively. For *in vitro* binding assays, 50 µl aliquot of beads with immobilized proteins were incubated for 60 min on ice with 1 µg of purified Hsp90 or Hsp90ΔMEEVD in reaction buffer (20 mM HEPES, pH 7.5, 20 mM KCl, 1 mM MgCl_2_, 0.01% NP-40) containing either no nucleotide, 5 mM ADP or 5 mM ATP. After incubation the supernatant was removed and the beads were washed thrice with the reaction buffer containing either no nucleotide, 5 mM ADP or 5 mM ATP. Proteins retained on the beads were extracted into SDS-sample buffer, separated by SDS-PAGE and immunoblotted with the R2 anti-Hsp90 antibody [33].

## Results

### Identification of CC-TPR proteins in *Arabidopsis*

Orthologs of most of the known CC-TPR co-chaperones of animal and yeast Hsp90 have also been identified in plants. To understand the extensiveness of CC-TPR proteins in the model plant *Arabidopsis*, the InterPro database was first searched for TPR proteins. The initial big list of 235 TPR proteins was narrowed down to 52 proteins on the criterion of proteins possessing a Hop TPR2a-like three-motif domain. The start and end sites of each of the three motifs in the 52 proteins were identified and the motifs were then searched for the conserved residues (K_5_N_9_-N_6_-K_2_R_6_) responsible for interacting with the MEEVD motif of cytosolic Hsp90. This led to the identification of 24 proteins, including SQUINT, which appeared in the database as a two-motif TPR domain. Following from this example, proteins showing one or two motifs of TPR were manually searched for the conserved residues, leading to another 12 proteins being identified as CC-TPR proteins with potential to interact with Hsp90/Hsp70. Thus, a total of 36 CC-TPR proteins were identified in *Arabidopsis* (Table 1). Analyses of these genes in TAIR, which provides structural and functional annotation as well as links to different databases containing information of specific gene, transcript, and/or protein, led us to infer that 24 of the 36 genes in the list were novel, while 12 genes had been characterized previously in *Arabidopsis* or another plant species (Table 1).

**Table 1:** Properties of the *Arabidopsis* CC-TPR proteins.

AGI ID	Name	Number of amino acids	Additional functional domains	Subcellular localization	mRNA species
**One TPR domain**					
AT4G30480	AtTPR1	161/208/277		C/N	3
AT1G04130	AtTPR2	360		C/*N*	1
AT1G04190	AtTPR3	328		C/N/*M*	1
AT1G04530	AtTPR4	310		C	1
AT1G56440	AtTPR5	476		N/P	1
AT1G58450	AtTPR6	164		*M*/C	1
AT5G21990	AtTPR7	554		M/N	1
AT4G08320	AtTPR8	426/427		C/*M*/N/P	2
AT1G33400	AtTPR9	798	SET	N	1
AT3G04710	AtTPR10	455/456	Ankyrin	*P*/C	2
AT2G25290	AtPhox1*	697	Phox/PB1	C/N	1
AT1G62390	AtPhox2*	751	Phox/PB1	*C*/M/N/*P*	1
AT5G20360	AtPhox3*	809	Phox/PB1	N	1
AT4G32070	AtPhox4*	811	Phox/PB1	*C*/N	1
AT4G22670	AtTPR11	441	Heat shock chaperonin-binding	**V**	1
AT2G15790	AtSquint	361	Cyclophilin	C	1
AT3G54010	AtPAS1	635/545	Peptidyl-prolyl-cis-trans isomerase	**C/N**	2
AT3G25230	AtROF1	551/562	Peptidyl-prolyl-cis-trans isomerase	C	2
AT5G48570	AtROF2	578	Peptidyl-prolyl-cis-trans isomerase	C/Pe	1
AT3G21640	AtTWD1	365	Peptidyl-prolyl-cis-trans isomerase	**PM**	1
AT3G07370	AtCHIP	278	U-box	C	1
AT2G42810	AtPP5	484/538	PP5	**C/N/ER**	2
AT3G17970	AtToc64-III	589	Amidase	**P/M**	1
AT5G09420	AtToc64-V	603	Amidase	**M**	1
**More than one TPR domain**					
AT1G78120	AtTPR12	530		**M**	1
AT5G10090	AtTPR13	594		*M*/N/P	1
AT5G65160	AtTPR14	593		M/*N*	1
AT2G41520	AtTPR15	1077/1108	DnaJ	P/N	2
AT5G12430	AtTPR16	1165	DnaJ	*P*/N	1
AT1G53300	AtTTL1	699	Thioredoxin	*N*/M/C	1
AT3G14950	AtTTL2	721	Thioredoxin	M/N/P	1
AT2G42580	AtTTL3	691	Thioredoxin	M/N/P	1
AT3G58620	AtTTL4	682	Thioredoxin	*P*/M/N	1
AT1G12270	AtHop1	572	Heat shock chaperonin-binding	*C*/N	1
AT1G62740	AtHop2	571	Heat shock chaperonin-binding	**PM**	1
AT4G12400	AtHop3	530/558	Heat shock chaperonin-binding	M/P/N	2

The 24 new CC-TPR proteins are referred to as AtTPR1-16, AtPhox1-4 and AtTTL1-4, while the known CC-TPR proteins are referred to by their names.

*represents new names of the proteins. For subcellular localization the bolded represent experimental documentation, while the italicized represent the most significant according to computational predictions. C, cytoplasm; ER, endoplasmic reticulum; M, mitochondria; N, nucleus; P, plastid; Pe, peroxisome, PM, plasma membrane; V, vacuole.

As an additional search of TPR proteins in *Arabidopsis*, we used the HMMER program, which aims to detect remote homologs on the basis of mathematical models, to generate a list of proteins against each motif of the CC-TPR proteins. The number of proteins generated against each motif varied and each list of proteins (data not shown) could be divided into four categories: 1) All 36 proteins described in Table 1 were among the top hits identified for all three motifs. An additional 2-6 proteins were detected in this list of proteins, which on analysis fell in the second category of proteins; 2) True TPR domain proteins (entry no. IPR013026), but lacking a three-motif TPR domain and/or the conserved carboxylate clamp residues. These proteins had been analysed earlier and rejected; 3) TPR-like domain containing proteins (InterPro entry no. IPR011990). The motif structure of this domain varied in the number of amino acids in different proteins and there was no conservation of the carboxylate clamp residues; 4) Proteins with no TPR domain. In conclusion, the HMMER search identified the same 36 CC-TPR proteins, lending further support to the results shown in Table 1.

Previously known CC-TPR proteins, such as Hop (AtHop1, AtHop2 and AtHop3), immunophilins (AtROF1, AtROF2, PAS1, AtTWD1 and SQUINT), AtCHIP, TOC64 (AtTOC64-III, and AtTOC64-V) and AtPP5 were all present in the list represented in Table 1. Hop, immunophilins, CHIP and PP5 are known co-chaperones [5], while Translocase of the mitochondrial outer membrane (Tom70), the functional homolog of TOC64, is a known interactor of Hsp90 in yeast and animal systems [34]. AtROF1and AtROF2 [19], AtTWD1 [21], and pea TOC64 [23] have been demonstrated to interact with Hsp90, while soybean Hop [15] and tomato PP5 [16] have been shown to act as co-chaperones of Hsp90. Of the newly identified proteins, TTL1 has been associated with salt sensitivity and altered abscisic acid (ABA) responses [35], but no links have been made of this protein to the Hsp90/Hsp70 chaperone machinery. The closest protein to TTLs in other organisms is Tpr2, a relatively new co-chaperone of mammalian Hsp90 that contains a DnaJ-like domain instead of the thioredoxin domain [35]–[37].

In addition to identifying members of a gene family, transcript data from NCBI also provides information on the number of splice variants for each gene family member. It is noted in Table 1 that there are two splice variants for PAS1, AtROF1, AtPP5, AtHop3, AtTPR8, AtTPR10 and AtTPR15, and three splice variants for AtTPR1. The DnaJ-domain containing AtTPR15 has two CC-TPR domains both of which are present in the first variant, but the second variant appears to be missing the first motif of the second TPR domain. With the exception of AtPP5 that has been shown to contain two splice variants localized in the cytoplasm/nucleus and ER, respectively [16], the splice variants of all other genes remain to be verified experimentally.

Multiple sequence alignments of excised TPR motifs of proteins listed in Table 1 against human Hop TPR2a as reference sequence showed high degree of conservation of the consensus residues K_5_N_9_-N_6_-K_2_R_6_ (Table 2). In the case of proteins with more than one TPR domain (AtTTLs, AtTPR12-16), the domain with highest conservation was used for alignment. It can be seen in Table 2 that with one exception, the substitutions for K_5_ and K_2_ in the first and third motif, respectively, are mostly conservative, whereby Lys (K) is replaced with Arg (R). Since this substitution occurs in confirmed Hsp90 interactors such as Hop, it is concluded that this substitution does not interfere with the binding of the TPR domain to Hsp90/Hsp70. The substitution of Asn (N_9_) with Gln (Q) in the first motif is also conservative. All others replacements are radical according to classification of amino acids by volume and polarity, with the K_5_ to Glu (E) replacement in AtTPR4 being most noteworthy. With the exception of AtPhox1 and AtPhox4, the consensus residue N_6_ of the second motif is highly conserved in *Arabidopsis* proteins. It should be noted that Ala (A) and Met (M) substitute R_6_ in the third motif of known co-chaperones AtHop1-3 and AtCHIP, respectively, indicating that some radical changes are accommodated in the TPR:Hsp90/Hsp70 interaction. How such substitutions affect interaction with Hsp90/Hsp70 remains to be seen.

**Table 2:** Multiple sequence alignment of excised TPR motifs of *Arabidopsis* proteins.

Name	AGI ID		Motif I		Motif II		Motif III
			Helix 1A Helix 1B		Helix 2A Helix 2B		Helix 3A Helix3B
Human Hop		225	ALKE**K**ELG**N**DAYKKKDFDTALKHYDKAKELDPTN	259	MTYIT**N**QAAVYFEKGDYNKCRELCEKAIEVGREN	300	A**K**AYA**R**IGNSYFKEEKYKDAIHFYNKSLAEHRTP
AtTPR1	AT4G30480	105	ANEA**K**AEG**N**KLFVNGLYEEALSKYAFALELVQEL	146	SICYL**N**RGVCFLKLGKCEETIKECTKALELNPTY	180	N**K**ALV**R**RAEAHEKLEHFEDAVTDLKKILELDPSN
AtTPR2	AT1G04130	31	AIEF**K**EEG**N**ECVRKGKKHYSEAIDCYTKAISQGV	71	SILFS**N**RSHVNLLLGNYRRALTDAEESMRLSPHN	105	V**K**AVY**R**AAKASMSLDLLNEAKSYCEKGIENDPSN
AtTPR3	AT1G04190	15	EKSL**K**EKG**N**EFFKAGNFLKAAALYTQAIKLDPSN	49	ATLYS**N**RAAAFLSLVKLSKALADAETTIKLNPQW	83	E**K**GYF**R**KGCVLEAMEKYEDALAAFEMALQYNPQS
AtTPR4	AT1G04530	136	PLLL**K**NYA***K***FLEYKGDLSGAEEYYHKCTVVEPSD	170	GVALA**N**YGRLVMKLHQDEAKAMSYFERAVQASPD	260	G***E***TLC**R**YAKAFWSINNDHEKALFYFEKAVEASPN
AtTPR5	AT1G56440	84	SSSE**K**EQG**N**EFFKQKKFNEAIDCYSRSIALSPNA	118	VTYAN***R***AMAYLKIKRYREAEVDCTEALNLDDRY	151	I**K**AYS**R**RATARKELGMIKEAKEDAEFALRLEPES
AtTPR6	AT1G58450	10	ANRK**K**EEG**N**LLYKTQKYERAAKKYNKAAECIENG	58	VSCFL**N**GAACSLKLKNFLETIVLCSEVLDIEFQN	92	V**K**ALY**R**RAQSYIEVGDLISAEMDINRALEADPEN
AtTPR7	AT5G21990	103	AQML**K**KQG**N**ELHSRGNFSDAAEKYLRAKNNLKEI	146	LACSL**N**LMSCYLKTNQHEECIKEGSEVLGYDARN	180	V**K**ALY**R**RGQAYRDLGLFEDAVSDLSKAHEVSPED
AtTPR8	AT4G08320	175	AETL**K**CQG**N**KAMQSNLYLEAVELYSFAIALTDKN	209	AVFYC**N**RAAAYTQINMCSEAIKDCLKSIEIDPNY	243	S**K**AYS**R**LGLAYYAQGKYAEAIEKGFKKALLLDPH
AtTPR9	AT1G33400	63	SLDL**K**RRG**N**HCFRSRDFDEALRLYSKALRVAPLD	106	ASLFL**N**RANVLHNLGLLKESLRDCHRALRIDPYY	140	A**K**AWY**R**RGKLNTLLGNYKDAFRDITVSMSLESSL
AtTPR10	AT3G04710	327	AAEA**K**ARG***Q***DAFHRKDFQMAIDAYTQAIDFDPTD	361	HTLFS**N**RSLCWLRLGQAEHALSDAKACRELNPDW	395	P**K**GCF**R**EGAALRLLQRFDEAANAFYEGVLLSPES
AtPhox1	AT2G25290	52	ALEL**K**EEG**N**KLFQKRDYEGAMFRYDKAVKLLPRD	90	AYLRT***S***MASCYMQMGLGEYPNAINECNLALEASP	126	S**K**ALL***K***RARCYEALNKLDFAFRDSRVVLNMEPEN
AtPhox2	AT1G62390	51	AHEL**K**EEG**N**KKFQARDYVGALEQYENGIKLIPKS	89	AVFHS**N**RAACLMQMKPIDYESVISECSMALKSQP	125	T***R***ALL**R**RARAFEAVGKFDLAVQDVNVLLGSDPNH
AtPhox3	AT5G20360	126	AQGL**K**EEG**N**KLFQKRDYDGAMFKYGEAIKILPKD	164	SHVRA**N**VASCYMQLEPGEFAKAIHECDLALSVTP	200	N**K**ALL***K***RARCYEALNKLDLALRDVCMVSKLDPKN
AtPhox4	AT4G32070	51	ALEL**K**EEG**N**KLFQKRDHEGAMLSFDKALKLLPKD	89	AYLRT***S***MASCYMQMGLGEYPNAISECNLALEASP	125	S**K**ALV**R**RSRCYEALNKLDYAFRDARIVLNMEPGN
AtTPR11	AT4G22670	123	AQEA**K**GKA***M***EALSEGNFDEAIEHLTRAITLNPTS	157	AIMYG**N**RASVYIKLKKPNAAIRDANAALEINPDS	191	A**K**GYK***S***RGMARAMLGEWAEAAKDLHLASTIDYDE
AtSquint	AT2G15790	212	VDFV**K**AHG**N**EHFKKQDYKMALRKYRKALRYLDIC	264	SQIFT**N**SAACKLKFGDAKGALLDTEFAMRDEDNN	298	V**K**ALF**R**QGQAYMALNNVDAAAESLEKALQFEPND
AtPAS1	AT3G54010	310	ADKI***R***STG**N**RLFKEGKFELAKAKYEKVLREFNHV	359	NMLHL**N**VAACLLKMGEWRKSIETCNKVLEAKPGH	393	V**K**GLY**R**RGMAYIAGGEYDDARNDFNMMIKVDKSS
AtROF1	AT3G25230	400	ASKK**K**EEG**N**SKFKGGKYSLASKRYEKAVKFIEYD	449	VACNL**N**DAACKLKLKDYKQAEKLCTKVLELESTN	483	V**K**ALY**R**RAQAYMELSDLDLAEFDVKKALEIDPNN
AtROF2	AT5G48570	410	AGKK**K**EEG**N**VLFKAGKYARASKRYERGVKYIEYD	459	IACNL**N**DAACKLKLKDYKEAAKLSTKVLEMDSRN	493	V**K**AMY**R**RAHAYLETADLDLAELDIKKALEIDPDN
AtTWD1	AT3G21640	179	ADRR**K**MDG**N**SLFKEEKLEEAMQQYEMAIAYMGDD	230	NPCHL**N**IAACLIKLKRYDEAIGHCNIVLTEEEKN	264	P**K**ALF**R**RGKAKAELGQMDSARDDFRKAQKYAPDD
AtCHIP	AT3G07370	10	AERL**K**EDG**N**NCFKKERFGAAIDAYTEAIALSPNV	44	PAYWT**N**RALCHMKRKDWTKVEEDCRKAIQLVHNS	78	V**K**AHY***M***LGLALLQKKEFTNGVKELQRALDLGRCS
AtPP5	AT2G42810	13	AEEF**K**SQA**N**EAFKGHKYSSAIDLYTKAIELNSNN	47	AVYWA**N**RAFAHTKLEEYGSAIQDASKAIEVDSRY	80	S**K**GYY**R**RGAAYLAMGKFKDALKDFQQVKRLSPND
AtToc64III	AT3G17970	474	AEIA**K**EKG**N**QAFKEKLWQKAIGLYSEAIKLSDNN	508	HVLFS**N**RSAAHASLNHYDEALSDAKKTVELKPDW	542	V**K**AYL**R**RGTAREMLGDCKGAIEDFRYALVLEPNN
AtToc64V	AT5G09420	488	SEVM**K**EKG**N**AAYKGKQWNKAVNFYTEAIKLNGAN	522	ATYYC**N**RAAAFLELCCFQQAEQDCTKAMLIDKKN	556	V**K**AYL**R**RGTARESLVRYKEAAADFRHALVLEPQN
AtTPR12	AT1G78120	159	PETL**K**KMG**N**EEYCRGRFGQALVFYERAISADPKT	193	PTYWP**N**KSAALISLGRLLEASDACEEALRLNPTY	227	E***R***AHQ**R**LASLQLRLGEVEKALCHYNEAGKYTETK
AtTPR13	AT5G10090	237	PETL**K**IMG**N**EDYKNGNFAEALALYEAAISIDPKK	271	ASYRS**N**KSAALTALGRILEAVFECREAIRIDPHY	305	H***R***AHH**R**LANLYLRLGEVENSIYHFKHAGPEADQE
AtTPR14	AT5G65160	470	VTEA***R***FKG**N**ELFKSGRFQEACAAYGEGLDHDPRN	504	SVLLC**N**RAACRSKLGQFDKSIEDCTAALSVRPGY	538	G**K**ARL**R**RADCNAKIEKWELAVGDYEILKKESPED
AtTPR15	AT2G41520	533	CEVW***R***LRG**N**QAYKNGYMSKAEECYTHGINSSPSK	599	ALCYG**N**RAAARISLGRLREAISDCEMAASLDPSY	631	I**K**AYM**R**AANCHLVLGELGSAVQYFNKCMKSTSSV
AtTPR16	AT5G12430	608	CEKW***R***LRG**N**NAYKIGDLSRAEESYTQGIDSVPRI	652	MLCYS**N**RAATRMALGRMREAIADCTMASSIDSNF	686	L**K**VQV**R**AANCYLSLGEIEDASRYFKKCLQSGSDI
AtTTL1	AT1G53300	465	VARA***R***ARG**N**DLYKSERYTEASSAYAEGLRLDPCN	499	AILYC**N**RAACWFKLGMWERSIEDCNQALRYQPSY	533	T**K**PLL**R**RAASNSKMERWGAAVSDYEALIRELPHD
AtTTL2	AT3G14950	258	PEEV**K**RFG**N**EMFRKGCFAEALKLYDRAIELSPSN	292	ATYHS**N**RAAALSSLGQIGEAVNECEIAIKLDPNF	326	A*R*AHH**R**LASLLLRLGYVDNAGIHLYSVEEPLDPT
AtTTL3	AT2G42580	458	VVRA***R***TRG**N**ELFSSGRFSEACVAYGDGLKQDDSN	492	SVLYC**N**RAACWYKLGLWEKSVEDCNHALKSQPSY	526	I**K**ALL**R**RAASYGKLGRWEDAVKDYEFLRRELPGD
AtTTL4	AT3G58620	449	VAKA***R***TRG**N**ELFSSGRYSEASVAYGDGLKLDAFN	483	SVLYC**N**RAACWFKLGMWEKSVDDCNQALRIQPSY	517	T**K**ALL**R**RAASYGKLGRWEDAVRDYEVLRKELPGD
AtHop1	AT1G12270	244	AKKE**K**ELG**N**AAYKKKDFETAIQHYSTAIEIDDED	278	ISYLT**N**RAAVYLEMGKYNECIEDCNKAVERGREL	323	T***R***KGT***A***LTKMAKCSKDYEPAIEAFQKALTEHRNP
AtHop2	AT1G62740	243	AQKE**K**ELG**N**AAYKKKDFETAIQHYSTAMEIDDED	277	ISYIT**N**RAAVHLEMGKYDECIKDCDKAVERGREL	322	T*R*KGT***A***LGKMAKVSKDYEPVIQTYQKALTEHRNP
AtHop3	AT4G12400	230	ALKE**K**GEG**N**VAYKKKDFGRAVEHYTKAMELDDED	264	ISYLT**N**RAAVYLEMGKYEECIEDCDKAVERGREL	309	T***R***KGS***A***LVKMARCSKDFEPAIETFQKALTEHRNP

The conserved residues are indicated in bold, and the substitutions in bold-italics. The numbers on the left of each motif refer to the amino acid positions in the sequences of proteins.

A notable feature of AtTPR5 is that the conserved N is detected in the fifth position of the second motif as opposed to the sixth position. If, however, the second motif is initiated at residue 117 instead of 118 in the protein sequence, then the N residue falls in the right place. The perfect match of residues in the first and third TPR motifs of AtTPR5 to the consensus K_5_N_9_ and K_2_R_6_, respectively, as well as the presence of a relatively conserved R residue after N in the second motif, suggest that AtTPR5 qualifies as a CC-TPR protein.

### *In silico* characterization of the newly identified CC-TPR proteins in *Arabidopsis*

**Figure 1:**
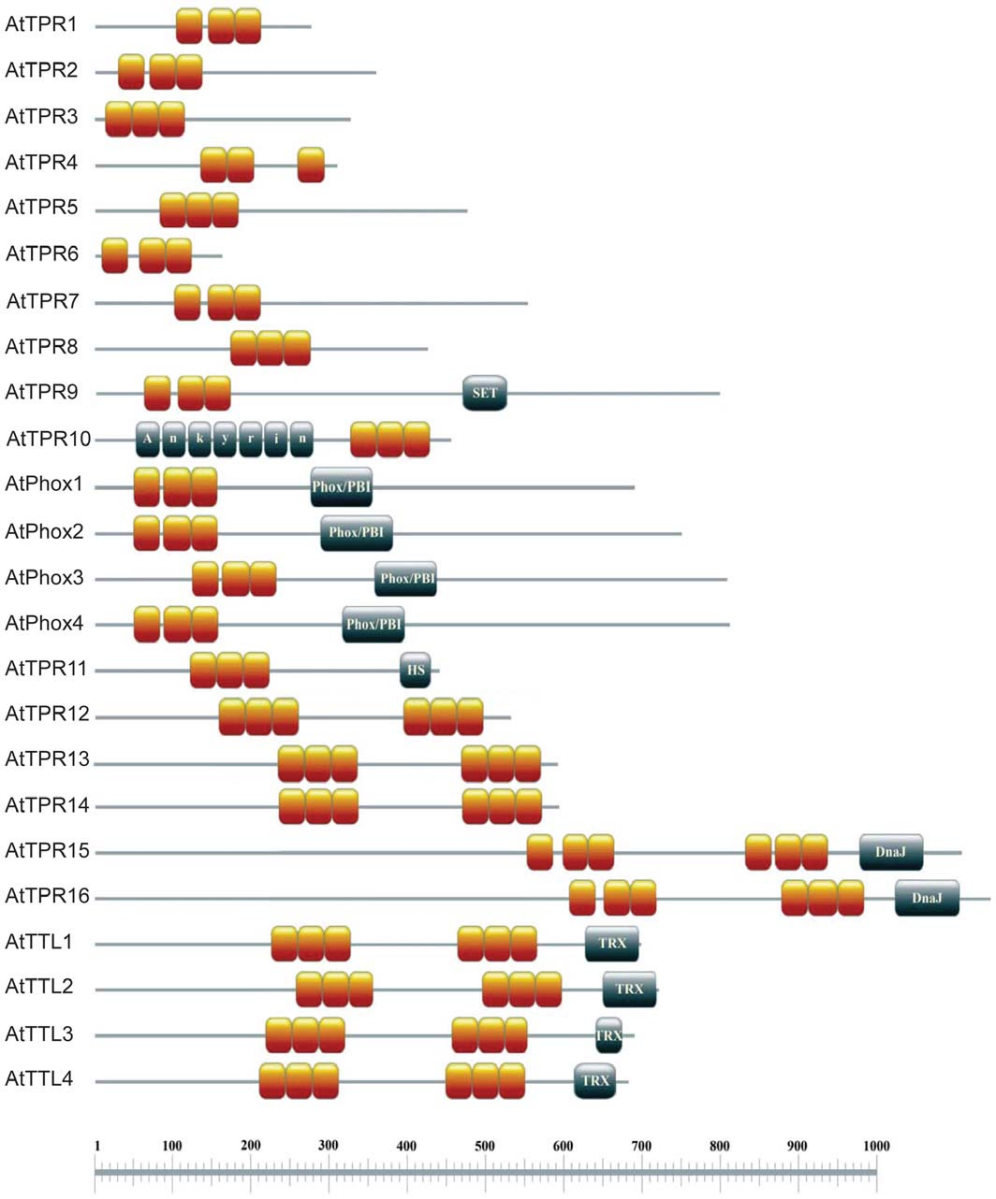
Domain schematics of the newly identified CC-TPR proteins in *Arabidopsis*. Each orange box represents one motif of the TPR domain, and the grey box represents another domain. The scale below indicates the size of the protein in number of amino acids. The schemes were generated using MyDomains from PROSITE (http://www.expasy.ch/tools/mydomains/).

To obtain clues about the functions of the newly identified CC-TPR proteins, InterProScan against InterPro database was used to identify additional functional domains as well as subcellular localization signal sequences within these proteins. The known functional domains were highlighted on the output display page for each input FASTA sequence. Using this information the schematics of domain architecture of the new proteins were prepared (Figure 1). Eight of the proteins contained a single TPR domain, seven contained a single TPR domain plus another functional domain, three contained two TPR domains, and six contained two TPR domains plus another functional domain. The second functional domains in these proteins include SET, ankyrin, octicosapeptide/Phox/Bem1p (PB1), DnaJ, and thioredoxin (Figure 1). With the exception of DnaJ, these domains have not previously been linked with CC-TPR proteins.

The ankyrin repeat is a protein-protein interaction motif comprised of a tandemly repeated 33 amino acid sequence. Each repeat folds into a helix-loop-helix structure [38]. Ankyrin repeats are present in functionally diverse proteins involved in transcription initiation, cell-cycle regulation, ion transportation and signal transduction. This domain was identified in AtTPR10, which is predicted to localize to the plastid (Table 1).

As a conserved sequence motif made up of 130–140 amino acids, the SET domain occurs as a part of multidomain proteins involved in histone methylation, which regulates chromatin structure and gene transcription [39]. These enzymes use *S*-adenosylmethionine (AdoMet) as a donor substrate and add methyl groups to lysine residues of histone H3. SET domain proteins can also methylate non-histone proteins [40]. Recently it was shown that a human protein called SMYD2 interacts with Hsp90α, and that this interaction enhances the histone methyltransferase activity of SMYD2 [41]. The presence of the SET domain in AtTPR9 and its predicted localization to the nucleus (Table 1) suggest that this protein may be involved in methylating histone or non-histone proteins.

AtPhox1 to AtPhox4 constitute a gene family (Figure S1). In addition to the TPR domain, the AtPhox proteins contain the Phox/PB1 domain (Figure 1), which is known to mediate protein-protein interactions in cell processes such as cell polarity, pheromone signaling, cytoskeletal organization, osteoclastogenesis, angiogenesis and early cardiovascular development [42]. Heterodimerization of two PB1 domains is important in the formation of macromolecular signaling complexes. PB1 domains can also interact with other protein domains. The presence of Ser (S) in place of the consensus N_6_ in the second TPR motif of AtPhox1 and AtPhox4 (Table 2) warrants investigation of this substitution in the context of Hsp90/Hsp70 binding.

The TTLs (AtTTL1-4) are a novel protein family (Figure S2) unique to plants that in addition to the TPR domain contain a motif in the C-terminus with homology to thioredoxins. The thioredoxin fold is involved in the regulation of protein activity by changes in the redox state of thiol groups (S_2_ to SH_2_) [43]. The localization of these proteins to plastid/mitochondria where redox reactions are common is consistent with their possessing a thioredoxin domain.

Co-presence of TPR and DnaJ domains in a CC-TPR protein has been noted before in the human Tpr2 co-chaperone [36]. Two of the newly identified CC-TPR proteins AtTPR15 and AtTPR16 contain the DnaJ domain, which mediates protein-protein interactions and various chaperone functions [44].

The heat shock chaperonin-binding motif found in Hop is for binding heat shock proteins. This domain is found singly or duplicated in proteins [45]. The newly identified protein AtTPR11 contains this domain. As would be expected, transcripts of proteins with this domain are induced by high temperature.

### Identification of CC-TPR proteins in rice

**Figure 2:**
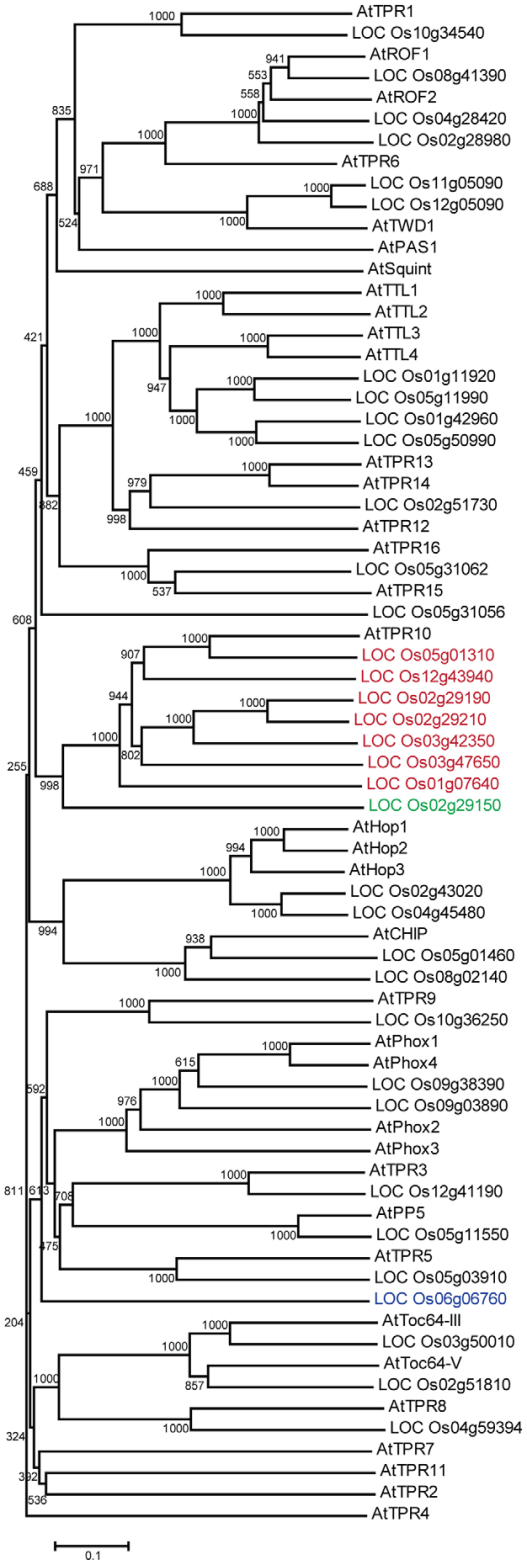
Phylogenetic tree showing sequence relationships between the CC-TPR proteins from rice and *Arabidopsis*. Full-length protein sequences were aligned using Clustal X 2.0.10. A phylogenetic tree was then derived using the neighbor-joining method in Clustal X. The numbers indicate bootstrap values based on 1000 replicates. The ankyrin containing proteins are highlighted in red, the protein with FBD and F-box is highlighted in green, and the protein with protein kinase (STYKc) and U-box is highlighted in blue.

The HMM profile created with *Arabidopsis* sequences was used to search for CC-TPR proteins in rice. The top hits for each of the motifs were searched for the conserved carboxylate clamp residues. Sequence alignments of each of the three motifs of the top scoring CC-TPR proteins in rice are shown in Table 3. In case of proteins with more than one three-motif TPR domain, the most conserved domain was used for alignment. Proteins with both conservative and non-conservative amino acid substitutions in the carboxylate clamp residues were identified. The protein sequences of CC-TPR proteins identified in *Arabidopsis* (Table 1) and rice (Table 3) were used to generate a phylogenetic tree (Figure 2). The various additional domains detected in *Arabidopsis* CC-TPR proteins were also found in rice CC-TPR proteins. In addition, we found that unlike in *Arabidopsis*, rice contains numerous ankyrin containing CC-TPR proteins (Figure 2, highlighted in red), one CC-TPR protein with FBD and F-box (highlighted in green), and one CC-TPR protein with protein kinase (STYKc) and U-box (highlighted in blue). The latter two proteins in rice likely expand the ability of the Hsp90/Hsp70 system to link cellular proteins to the ubiquitin proteasome system.

**Table 3:** Multiple sequence alignment of excised TPR motifs of rice proteins.

Locus ID		Motif 1		Motif 2		Motif 3
Os10g34540	64	ANDA**K**AEG**N**KFFGAGEYERALSQYETALQIAAEL	105	SACHS**N**RAVCFLKLGKYDETIKECTKALELNPSY	139	L**K**ALL**R**RGEAHEKLEHYDEAIADMKKIIELDPSN
Os12g41190	14	SAAL**K**DQG**N**EQFKAGNYLKAAALYTQAIKLDPDN	48	PTLYS**N**RAAAFLHLVKLNKALADADTTIKLKPQW	82	E**K**GHF**R**KGCVLESMEHYEEAISSFQIALQHNPQN
Os05g03910	40	AASE**K**EQG**N**EYFKQKKFAQAIECYSRSIGLSPSA	74	VAFA**NR**AMAYLKLRRFEEAENDCTEALNLDDRY	107	V**K**AYS**R**RITARKELGKLKEAMDDAEFAVSIDPNN
Os04g59394	181	AEFF**K**SKG**N**EFMRSKQHLKAVELYTCAIALSRNN	215	AIYYC**N**RAAAYTLLNMFNEAVEDCLKSIEIDPNY	249	S**K**AYS**R**LGSAYFALGKYHDALYKGYLKASELDPS
Os10g36250	63	AAEL**K**GKG**N**ACFSKREFEQALGFYSQALRYFPIS	106	ATLYV**N**RASTMHKLGLLEECLRDCDRAISVSPNY	140	A**K**AWY**R**RGMVNASFRNYSSSIHDLEVALSMEVTS
Os05g01310	329	SLEA**K**SRG***D***DAFRNKDYLVAVDAYTQAIELNPND	363	ATLHS**N**RSLCWLRAGQAERALEDARACRALRPDW	397	A**K**ACY**R**EGAALRLLQRFEEAANAFYEGVQLEPEN
Os12g43940	323	RSDM**K**QQG***D***AAFKKQDYLNASVFYTQALKVDPFD	357	GTLFS**N**RSLCWLRMGDGERALDDANACEKLRPKW	391	A**K**SYY**R**QGAALMFLKEYERAHRALGRALELDPES
Os02g29190	228	ATDL**K**SLG**N**KAVEKKDYLSATGFYSKALYLYPDD	262	ATLFS**N**RSLCWHRMGDGGKALLDAHECRKLRSDW	296	P**K**AYY**R**LGAALMLLKDYESACEALYNGFKLDPGN
Os02g29210	247	ATEL**K**SLG**N**KAVEKKDYLSATGFYSKALDLYPDD	281	ATLFS**N**RSLCWHHMGNGGKALLDAYECRKLRPDW	315	P**K**AYY**R**QGAALMLLKDYESACETLYDGLKLDPGN
Os03g42350	273	IAEF**K**SLG***L***EAVEKKDYLSAAGFYSEAMDLDPDD	307	ATLLS**N**RSLCWLYLGEGGKALVDAHKCRKMRPDW	341	P**K**ACY**R**QGAALMLLKDYVSACEALFDGFKLDPED
Os03g47650	343	KAQL**K**SLG***A***SAVQGKDYVGASKFYSEAIQLDPTD	377	ATLHS**N**RSFCYLKSGEAREALVDAKTCIGLKPDW	411	P**K**GYY**R**KGAALMSLKEYKEACDAFMDGVKLDPAS
Os01g07640	318	KDEL**K**LQG**N**SSFNNEDYDAAILLYSMAMKFDNTD	352	AKLYS**N**RSACWLNLGIGDEALSDAQICSKMQPDW	386	A**K**GYY**R**QGMAFSLLQDYASASYVLRRALKLDPQN
Os09g38390	45	AQEL**K**EEG**N**KLFQRREHERALLNYEKAIKLLPRG	83	AYLHS**N**LAACYMQMSPPDHYRAINECNLALDASP	119	S**K**ALL***K***RARCFEALGRLDLAYRDVAKVLAVEPNN
Os09g03890	53	AIEL**K**DEG***T***RLFQRRDYEEAAIKFGEAIKLLPKE	91	AFLHC**N**AAACYMHMNPEDLDHAIEECNLALEASP	127	T**K**ALL***K***RARCFEALDKLDLACKDVQKVLSLEPSN
Os08g41390	406	AGAK**K**EEG**N**ALFKLGKYVRASKRYEKAAKFIEYD	455	VTCNL**N**NAACKLKLKDYKQAEKLCTKVLELDSQN	489	V**K**ALY**R**RAQAYMQLADLELAEVDIKKALEIDPDN
Os04g28420	411	AAKK**K**DEG**N**VWFKMGKYAKASKRYEKAAKYIEYD	460	VSCKL**N**NAACKLKLKEYREAEKLCTKVLELESTN	494	V**K**ALY**R**RTQAYIELADLELAELDVKKALEIDPDN
Os02g28980	456	AAKK**K**DEG**N**AWFKMEKYARASKRYGKALNFIQYD	505	VSCKL**N**NAACKLKLKDYKEAKELCTEVLELDSMN	539	V**K**AFY**R**RAQAHMYLVDFDLAELDIKKALEIDPDN
Os11g05090	191	ADRR**K**IEG**N**EYFKEKKFEEAMQQYEMAIAYMGDD	242	NPCHL**N**MAACLIKLKRFDEAIAQCSIVLAEDENN	276	V**K**ALF**R**RGKARAELGQTESAREDFLKAKKHSPED
Os12g05090	186	ADRR**K**IEG**N**EYFKEKKFEEAMQQYEMAIAYMGDD	237	NPCHL**N**MAACLIKLKRFDEAIAQCTIVLSEDENN	271	V**K**ALF**R**RGKARAELGQTESAREDFLKAKKYSPED
Os05g01460	68	AELR***R***IEG**N**SCFNKARLGAAIDCYTEAIALCPDV	102	AVYWL**N**RGLCHFKRKEWAKVEEDSRRALALDDTL	136	V**K**GHY***L***LGCAMLEKEQCALAIKEFNKALDLLKSS
Os08g02140	14	AELL**K**QEG**N**AFFKKDRISAAIDAYTGAIALCPKV	48	AVYWT**N**RALCYKRRNEWVRAEEDCRTAIQLDSHS	82	V**K**AHY***M***LGLALLNKDELAEGIKELEKSLELGRGA
Os06g06760	154	ADHH***R***DRG**N**DFFKQKRYQEAAMHYTEAMKKNPKD	188	PRVFS**N**RAQCHIYLGALPEGLEDADKCIALDPTF	222	L**K**GYL**R**KAKVQLLMGNYEIALATYVEGLKCDPNN
Os05g11550	12	SEEL**K**LKA**N**DAFKANKFSLAIELYSQAIELNSSN	46	AVYWA**N**RAFAHTKLEEYGSAVQDASKAIEIDARY	80	S**K**GYY**R**RGAAYLAMGKFKEALKDFQQVKRISPND
Os03g50010	470	AEAA**K**EKG**N**IAFKEKQWQKAINFYTEAIKLNNKV	504	ATYYS**N**RAAAFLELASYRQAEADCTSAIDIDPKI	538	V**K**AYL**R**RGTAREMLGYYKEAVDDFSHALVLEPMN
Os02g51810	497	AELL**K**EKG**N**SAFKGRKWSKAVEFYSDAIKLNGTN	531	ATYYS**N**RAAAYLELGRYKQAEADCEQALLLDKKN	565	V**K**AYL**R**RGIAREAVLNHQEALQDIRHALALEPQN
Os02g51730	225	PEKL**K**EMG**N**EEYREGHYAEAVALYDQAIMVDPTR	259	PAYWS**N**KAAALAALGRLIEAVGDCREAVRIDPSY	293	G***R***AHH**R**LGGLYLRLGEPDKAIHHFKQSANDSTGA
Os05g31062	98	LLSH**K**AAG**N**EAFQARRYSEAVEQYSAALARNSDS	136	AVCFC**N**RAASYQALGQVTDAIADCSLAMVLDATY	170	L**K**AIS**R**RATLYEMIRDYGQAANDLRKLISLIEKQ
Os05g31056	538	CETW***R***TSG**N**QAYTNGHFATAEEYYTRGINSVSGH	582	MLCYS**N**RAATRMSLGRMREALQDCLIATSIDPTF	616	L**K**AKV**R**AANCQLALGDLEDALRSYTACLTSSKTS
Os01g11920	438	VARA***R***SLG**N**ELFNSGKFSEACLAYGEGLKHHPVN	472	PVLYC**N**RAACRFKLGQWEKSIEDCNEALKIQPNY	506	P**K**ALL**R**RAASYGKMERWAESVKDYEVLRKELPGD
Os05g11990	247	VAQA***R***TLG**N**ELFHSGKFAEAFLAYGEGLKHHPAN	281	SVLYC**N**RAACMFKLGQWEKSIEDCNEALKIQPNY	315	W**K**ALL**R**RAASYGKIEQWADSVKDYEVLRRELPGD
Os01g42960	215	VAKA***R***AQG**N**ELYKAAKFSDASIAYSEGLKYEPSN	249	PVLYC**N**RAACWGKLERWEKAVDDCNEALRIQPNY	283	T**K**ALL**R**RASSYAKLERWADCVRDYEVLHKELPAD
Os05g50990	218	LQEV***T***RAG**N**EWYKKGHYGEALRHYDQAVALCPDS	252	AACRS**N**RAAALIGLGRLAEALRECEEAIRRDPAS	286	G***R***AHS**R**LAALCLRFGMVERAREHFMLAGQVNQSD
Os02g43020	2	ADEA**K**AKG**N**AAFSAGRYEEAARHFTDAIALAPGN	36	HVLYS**N**RSAALASVHRYSEALADAEKTVELKPDW	70	A**K**GYS**R**LGAAHLGLGDAASAVAAYEKGLALDPTN
Os04g45480	2	ADEA**K**AKG**N**AAFSAGRFEEAAAHFTDAIALAPDN	36	HVLYS**N**RSAAYASLHRYPEALADAERTVALRPDW	70	A**K**GCS**R**LGAARLGLGDAAGAVAAYEKGLALEPSN
Os02g29150	533	ATEL**K**SLG**N**KAVEKKDYLSATGFYSQAVDLYPDD	567	ATLFS**N**RSLCWHHMGDGHKALLDAYECRKLRPDW	601	L**K**AYY**R**QGAALMLLKDYESACETLYDGFKLDPGN

The conserved residues are indicated in bold, and the substitutions in bold-italics. The numbers on the left of each motif refer to the amino acid positions in the sequences of proteins.

### Expression patterns of CC-TPR proteins in *Arabidopsis*

A collaborative microarray project (based on Affymetrix ATH1 arrays), dubbed AtGenExpress, has utilized 79 different *Arabidopsis* samples in triplicate to generate expression data [29], [30]. We rationalized that the expression patterns of Hsp90 co-chaperones must overlap to some degree with the expression patterns of Hsp90 family members. Data to this end could help formulate functional hypotheses for these proteins in the context of the Hsp90/Hsp70 chaperone machinery. We first performed e-Northern analysis with the Expression Browser at BAR using the AtGenExpress extended tissue and stress series data sets for the *AtHsp90* gene family. The *Arabidopsis Hsp90* gene family members are referred to as *AtHsp90-1* (*AT5G52640*), *AtHsp90-2* (*AT5G56030*), *AtHsp90-3* (*AT5G56010*), *AtHsp90-4* (*AT5G56000*), *AtHsp90-5* (*AT2G04030*), *AtHsp90-6* (*AT3G07770*), and *AtHsp90-7* (*AT4G24190*). AtHsp90-1 to AtHsp90-4 are cytoplasmic, AtHsp90-5 is plastidial, AtHsp90-6 and AtHsp90-7 are localized to the mitochondria and ER, respectively [10]. Of these, *AtHsp90-3* was not present on the ATH1 array and is therefore missing from our analysis.

**Figure 3:**
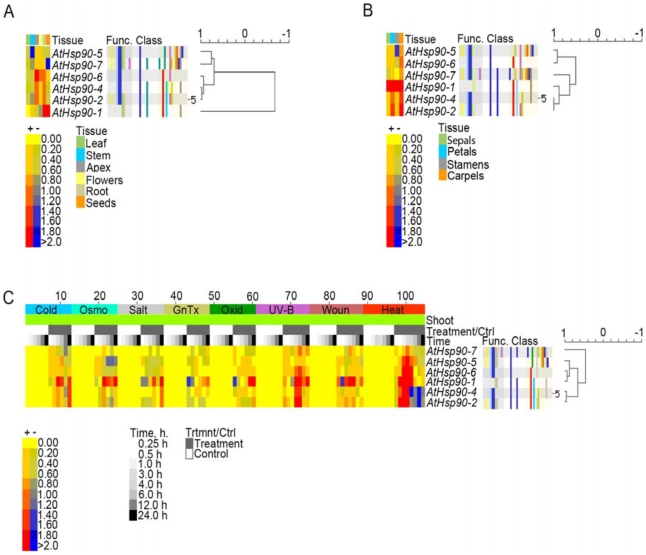
Expression profiles of *AtHsp90* genes. (A) e-Northern results for expression of *AtHsp90* genes in root, stem (second internode), leaf (cauline leaves), apex (shoot apex; inflorescence), flowers (stage 12), and seeds (stage 10, without siliques). (B) e-Northern results for expression of *AtHsp90* genes in individual floral organs (flowers stage 12), sepals, petals, stamens and carpels. (C) e-Northern results for expression of *AtHsp90* genes in response to different abiotic stresses. The control is mock treatment at each time point. The colour scale indicates the log_2_-level of expression above or below the median. Dark red indicates more than 4-fold above the median, while dark blue indicates 4-fold below. The clustering tree can be seen to the right of the heatmap.

When absolute expression values retrieved by AVT were used to compare the expression levels of *AtHsp90* gene family members, *AtHsp90-4* was found to have the highest level of constitutive expression, and *AtHsp90-1* to be most responsive to HS (data not shown). For e-Northern data from BAR depicting tissue expression, the median level of expression across all samples displayed for a particular gene is used as control value for calculating the relative level. Data in Figure 3A shows that with the exception of *AtHsp90-1*, which is highly expressed in seeds, most other members have higher expression in the shoot apex, followed by roots and flowers. Further dissection of the flower into sepals, petals, stamens and carpels revealed that in general carpels and petals have higher levels of *AtHsp90* transcripts as compared to the other two floral organs (Figure 3B). *AtHsp90* genes are induced by various abiotic stresses, such as cold, salt, UV-B, etc., although maximum induction is in response to HS (Figure 3C). These data are in accordance with our earlier observations in *Brassica napus* of the relatively high expression of *Hsp90* in apex, flowers and seeds, as well as of its responsiveness to cold temperatures [46].

**Figure 4:**
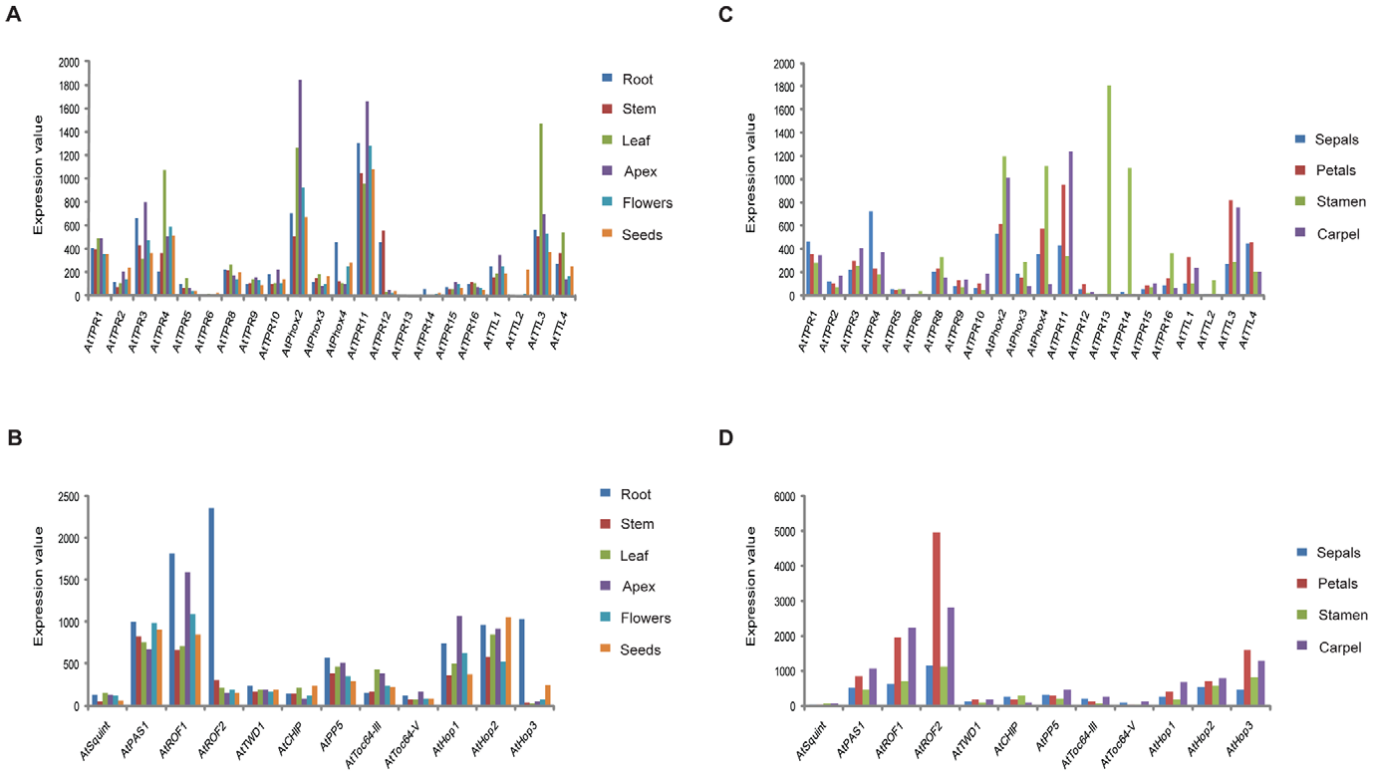
Expression profiles of *Arabidopsis CC-TPR* genes in different plant parts. Absolute expression values were retrieved by AVT and plotted as such. The developmental stage with the highest expression value was used for each transcript. Expression profiles of new *CC-TPR* genes (A) and of known CC-TPR co-chaperones (B) for root, stem, leaf, apex, flowers and seeds. Expression profiles of new *CC-TPR* genes (C) and of known CC-TPR co-chaperones (D) for individual floral organs: sepals, petals, stamens and carpels.

**Figure 5:**
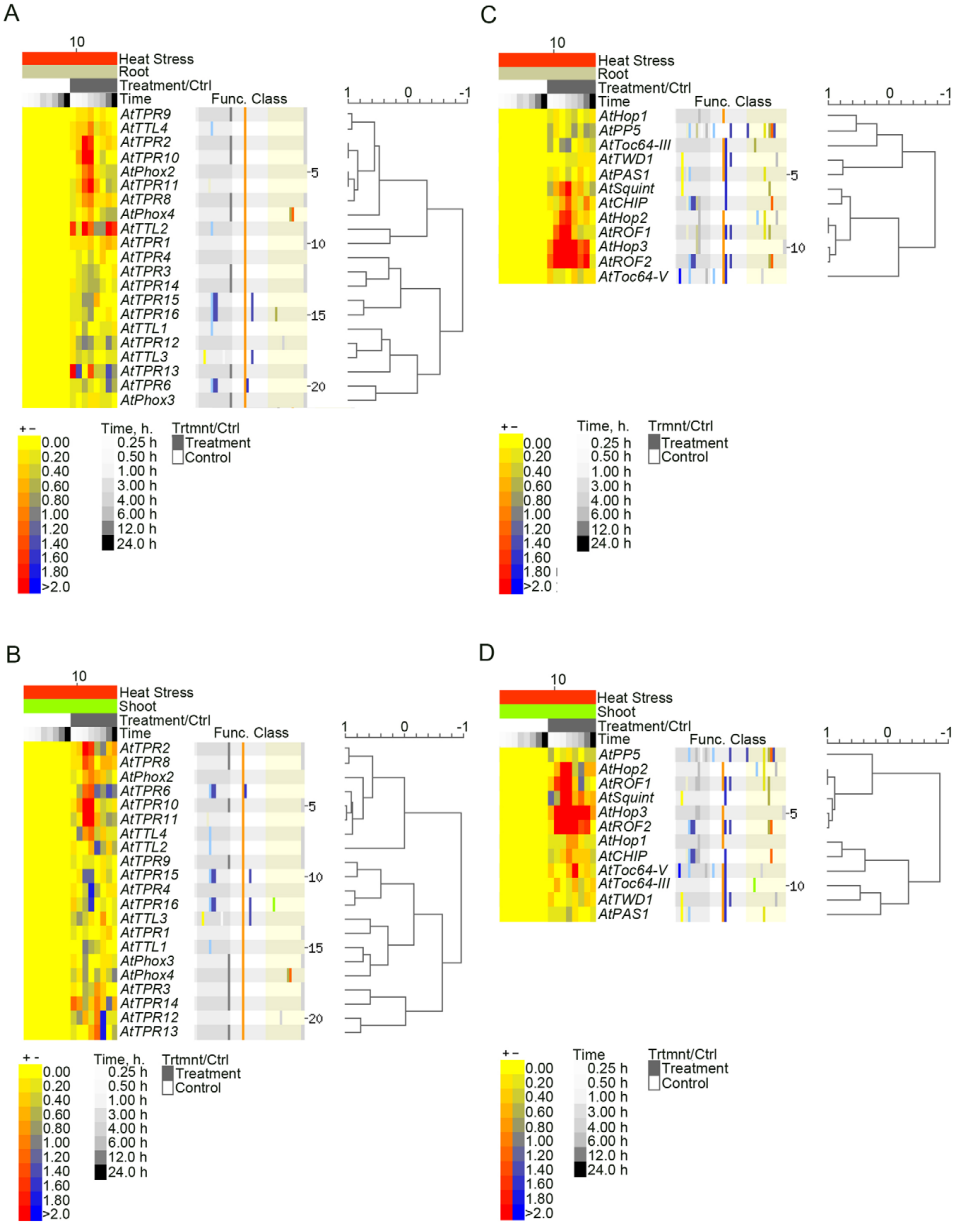
Expression profiles of *Arabidopsis CC-TPR* genes in response to HS. e-Northern results for expression of new *CC-TPR* genes in roots (A) and shoots (B) of seedlings exposed to HS. e-Northern results for expression of known CC-TPR co-chaperones in roots (C) and shoots (D) of seedlings exposed to HS.

**Figure 6:**
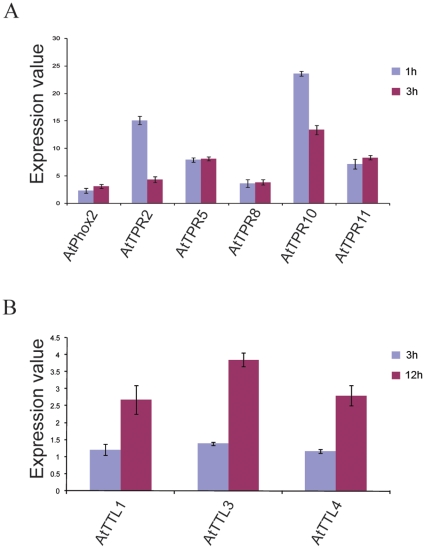
Transcript expression analysis by qRT-PCR in response to HS and BR treatment. (A) For HS treatment, 10 day-old *Arabidopsis* seedlings were exposed to 38°C for 1 and 3 h at which time the plant tissue above the medium was collected and quick-frozen for RNA isolation. (B) BR treatment was given to 21 day-old seedlings for 3 and 12 h and the tissue was collected as in A. Transcripts were analyzed by qRT-PCR. Bars indicate mean ± SD.

To compare the expression levels of *CC-TPR* genes (*AtTPR7* and *AtPhox1* were not present on the ATH1 array) in different plant parts, the absolute expression values retrieved by AVT were plotted (Figure 4). This was done because the low level expression of some *TPR* genes led to artifacts in the output of the Expression Browser. Several conclusions can be drawn from the data represented in Figure 4: 1) most genes are expressed in different plant parts, albeit at much lower levels than the highly expressed *AtHsp90-4*; 2) of the newly identified genes, *AtTPR11* and *AtPhox2* are expressed at relatively high levels in most tissues (Figure 4A), suggesting that the encoded proteins may be general co-chaperones of Hsp90/Hsp70; 3) of the known co-chaperones, immunophilins (*AtPAS1*, *AtROF1* and *2*) and Hop (*AtHop1, 2* and *3*), are expressed at relatively high levels (Figure 4B) and, like their animal counterparts, are abundant co-chaperones of plant Hsp90. The near to exclusive expression of *AtTPR13* and *AtTPR14* in stamens, and the relatively high expression of *AtPhox* genes, *AtTPR16*, *AtTTL2*, *AtTPR6* and *AtTPR*8 in stamens as compared to other floral organs (Figure 4C) is noteworthy. The immunophilins and *AtHop*, similar to *AtHsp90*, have predominant expression in petals and carpels (Figure 4D). Together, these data indicate that the Hsp90/Hsp70 chaperone machinery components are expressed in all plant tissues, albeit at different levels. It is possible that some components may function as general co-chaperones, while others may have tissue-specific functions within the context of the Hsp90/Hsp70 chaperone machinery. The expression profiles of the newly identified CC-TPR proteins provide an initial working hypothesis for delineating their functions.

e-Northern analysis of *CC-TPR* genes by BAR and AVT using the stress series data set indicated that heat is the most prominent signal for induction of transcript abundance. *AtTPR2*, *AtTPR5* (by AVT), *AtTPR8*, *AtTPR10, AtPhox2* and *AtTPR11* are heat-responsive in both root and shoot (Figure 5A, B). The fold-change values for *AtTTL2, AtTPR13* and *AtTPR14* in the output of Figure 5 are unreliable due to their low expression; these genes are therefore not considered as heat-responsive. *AtROF1, AtROF2, AtHop2*, *AtHop3* and *AtSquint* are also heat-induced both in root and shoot (Figure 5C, D), an attribute that has been noted before [15], [19]. The heat-induced expression patterns of *CC*-*TPR* genes are similar to that of *AtHsp90-1*. This observation supports a co-chaperone role for the encoded proteins during HS. The co-expression of this subset of genes with *AtHsp90-1* was confirmed using Expression Angler. *AtHsp90-1* was used as query in AtGenExpress stress series, and the data generated by Expression Angler was searched for the presence of *CC-TPR* genes during HS. *AtROF1*, *AtROF2*, *AtHop2*, *AtHop3*, *AtTPR2, AtTPR10 and AtTPR11* were within the top 156 proteins that are HS-induced and co-expressed with *AtHsp90-1* (data not shown). As a final confirmation, the HS response of *AtTPR2*, *AtTPR5*, *AtTPR8*, *AtPhox2*, *AtTPR10* and *AtTPR11* in leaf tissue was checked by qRT-PCR. All of these genes showed induction of expression in response to heat (Figure 6A).

Analysis of the AtGenExpress data set revealed some interesting information. One was the stress and ABA-responsive expression of the *AtPhox* gene family. *AtPhox2* was found induced by heat (Figure 5), *AtPhox3* by cold and salt stress, and *AtPhox4* by cold, osmotic and salt stress, as well as pathogen infection. In accordance with the role of ABA in stress tolerance [47], *AtPhox3* and *4* were found to be ABA-responsive (data can be retrieved by AVT). The second interesting observation was the brassinosteroid (BR) responsive expression of the *AtTTL* gene family. Three out of four *TTL* genes that have wide spread tissue expression, were found to be most responsive to BR as compared to other hormones. Since BR is a relatively new hormone [48], [49], the list of BR-regulated genes is still growing. To add *AtTTL* genes to this list we confirmed the BR-responsive expression of *AtTTL1*, *AtTTL3* and *AtTTL4* in *Arabidopsis* seedlings by qRT-PCR. All three genes showed 2 to 3-fold induction by BR at 12 h (Figure 6B). These results strongly suggest that *AtTTL1*, *3* and *4* are BR response genes.

### Expression patterns of CC-TPR proteins in rice

**Figure 7:**
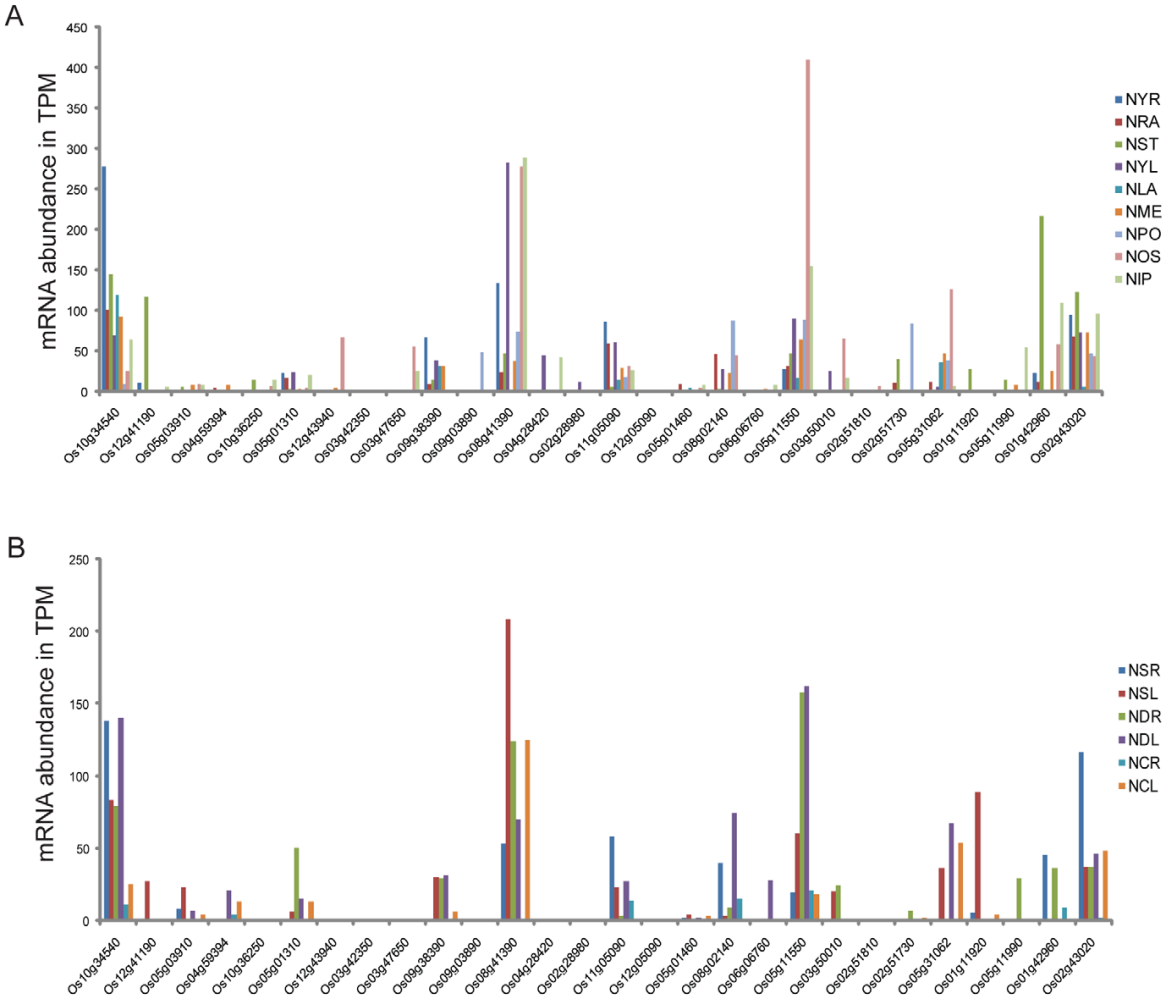
Transcript abundance of rice *CC-TPR* genes in tissue-specific and salinity, drought and cold -specific MPSS libraries. (A) Transcript abundance in NYR (young roots, 14 days); NRA (mature roots, 60 days) replicate A; NST (mature stem, 60 days); NYL (young leaves, 14 days); NLA (mature leaves, 60 days) replicate A; NME (crown vegetative meristematic tissue, 60 days); NPO (mature pollen); NOS (ovary and mature stigma); and NIP (immature panicle, 90 days). (B) Transcript abundance in NSR (young roots, 14 days) and NSL (young leaves, 14 days) stressed by 250 mM NaCl for 24 h; NDR (young roots) and NDL (young leaves) stressed by drought for 5 days; NCR (young roots) and NCL (young leaves) stressed by 4°C cold for 24 h.

We searched tissue-specific and stress-related libraries in the rice MPSS database (http://mpss.udel.edu/rice/) using 17 nucleotides long signatures to obtain information on the relative transcript abundance of rice *CC-TPR* genes. Of the 35 rice *CC-TPR* genes being reported here, no expression was detected for seven genes (Os02g29190, Os02g29210, Os01g07640, Os05g31056, Os05g50990, Os04g45480 and Os02g29150) in the tested conditions. Genes that were widely expressed (several tissues) at relatively high levels (Figure 7A) and most strongly induced by stress (salinity, drought and cold) (Figure 7B) are Os10g34540 (AtTPR1), Os08g41390 (AtROF1), Os05g11550 (AtPP5), and Os02g43020 (AtHop). The most closely related *Arabidopsis* proteins are given in parentheses. Tissues that showed highest transcript abundance in general are ovary and mature stigma (NOS), stem (NST), immature panicle (NIP), young leaves (NYL), and young root (NYR). Since we had observed a subset of *Arabidopsis CC-TPR* genes to be expressed exclusively or at the highest level in stamens, we paid close attention to the rice genes expressed similarly in pollen. Based on maximal expression of Os02g51730 (closest to AtTPR13/AtTPR14, Figure 2) in pollen as compared to its expression in other tissues (Figure 7A), and the same of AtTPR13/AtTPR14 in stamens (Figure 4C), it is tempting to speculate that Os02g51730 is a functional ortholog of AtTPR13/AtTPR14. Similarly, salt and drought -responsive expression of Os01g11920, Os05g11990 and Os01g42960 (Figure 7B), together with their sequence and structural similarities with AtTTLs (Figure 2), suggest that these proteins are functional orthologs of each other. The protein with the unique combination of CC-TPR, protein kinase, and U-box domains (Os06g06760) was found expressed at low levels in immature panicle and to be induced by drought stress.

### AtTPR1 and AtTPR2 interact with Hsp90 by the molecular ‘clamp’ mechanism

**Figure 8:**
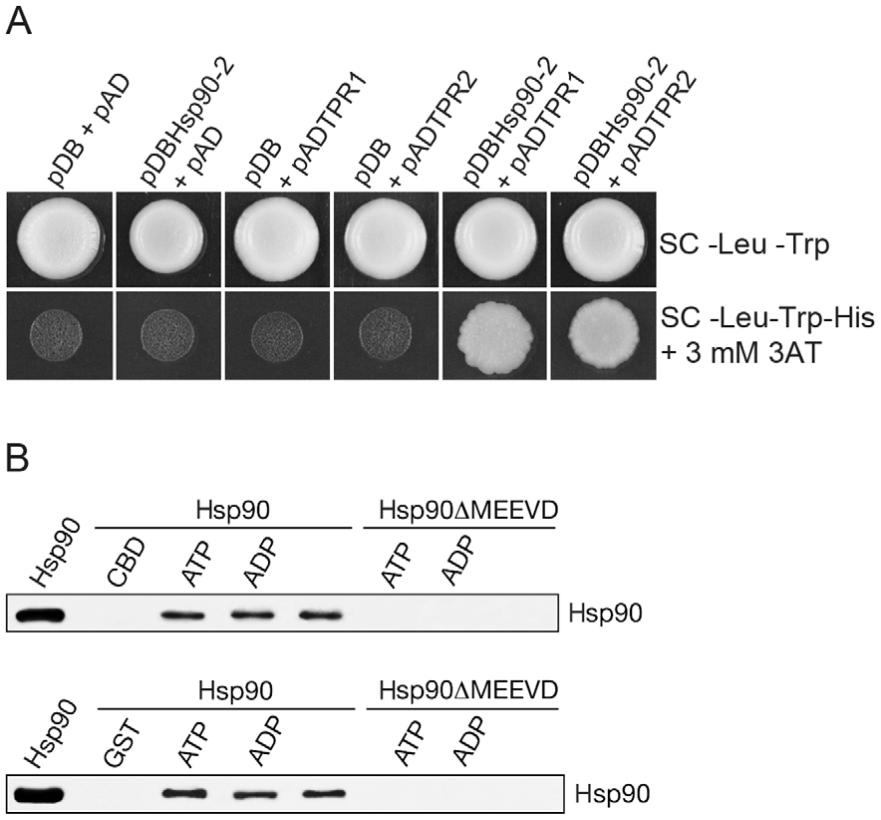
Interaction of AtTPR1 and AtTPR2 with Hsp90. (A) Positive interaction between AtHsp90-2 and AtTPR1/AtTPR2 in yeast, resulting in the activation of reporter genes, was detected by growth on SC –Leu –Trp –His + 3 mM 3AT (lower panel). No growth on this medium was observed for plasmid combinations (as indicated in the figure) when either AtHsp90-2 or AtTPR1/AtTPR2 or both were absent. Yeast cells were grown on SC –Leu –Trp to select for both pDB and pAD plasmids (upper panel). (B) *In vitro* binding of recombinant Hsp90 or Hsp90ΔMEEVD to immobilized TPR1-CBD (upper panel) and TPR2-GST (lower panel) in the absence of any nucleotide or in the presence of 5 mM ADP or 5 mM ATP. After formation of complexes, washing of the beads and elution of proteins, Hsp90 was detected by immunoblotting. An aliquot of purified Hsp90 was run on the gel to mark the position of Hsp90 (extreme left). Controls were immobilized CBD and GST incubated with Hsp90.

We applied yeast two-hybrid and *in vitro* binding assays as a proof-of-concept trial for analysis of the newly identified proteins for binding to Hsp90. AtHsp90-2 fused to the DNA-binding domain (pDBHsp90-2) and AtTPR1/TPR2 fused to the activation domain (pADTPR1/TPR2) were co-expressed in yeast. Growth of yeast on selection medium when co-transformed with pDBHsp90-2and pADTPR1/TPR2 only, but no other plasmid combination (Figure 8A), indicated positive interaction between Hsp90 and AtTPR1/TPR2. This interaction was further validated in *in vitro* binding assays. TPR1-CBD and TPR2-GST immobilized on chitin and Glutathione Sepharose 4B beads, respectively, were incubated with purified recombinant AtHsp90-2 in the absence or presence of ATP and ADP. Both TPR1-CBD (Figure 8B, top panel) and TPR2-GST (bottom panel) bound Hsp90 in a nucleotide independent manner, but no binding was seen to CBD or GST alone. Neither TPR1-CBD nor TPR2-GST bound to Hsp90ΔMEEVD, confirming that the interaction requires MEEVD for carboxylate clamp formation. These results provide evidence for the validity and success of our *in silico* strategy of identifying TPR proteins with potential to interact with Hsp90/Hsp70.

## Discussion

### Identification of new CC-TPR proteins in *Arabidopsis* and rice by *in silico* methods

Co-chaperones are an integral part of the Hsp90/Hsp70 chaperone protein folding machinery, which has key roles in numerous cellular processes [1]–[5]. The known CC-TPR co-chaperones (Hop, immunophilins, PP5, CHIP, TOM70, Tpr2) bind to the MEEVD motif of Hsp90 and regulate the functions of Hsp90 [5], [8]. Orthologs of these are present in plants, and mutations in some have shown striking phenotypes. Due to the significance of the CC-TPR co-chaperones in the biochemical regulation of the Hsp90/Hsp70 machinery, we set out to uncover all CC-TPR proteins encoded by the model plant *Arabidopsis* genome based on the information available through studies in animal and yeast systems. The confirmation of two of the 24 newly identified CC-TPR candidates to bind to the MEEVD motif of Hsp90 indicates a high probability for these proteins as bona fide interactors of either Hsp90 or Hsp70 or both. This data set provides a useful framework that will accelerate studies of the Hsp90/Hsp70 chaperone machinery in plants, as well as of the newly identified CC-TPR proteins, most of which have no assigned functions.

To predict the CC-TPR co-chaperone network in *Arabidopsis*, sequences with the well characterized protein interaction domain called ‘TPR’ were extracted from the InterPro database, followed by careful identification of the conserved residues involved in binding with the MEEVD motif of Hsp90 [8]. Within this protein set, 12 proteins are orthologs of previously known co-chaperones of Hsp90/Hsp70, and 24 are new proteins with potential to serve as co-chaperones of Hsp90/Hsp70. A similar search in rice identified 35 CC-TPR proteins. The presence of all CC-TPR proteins (GmHop, AtROF1, AtROF2, AtTWD1, AtCHIP, AtSQUINT, LePP5 and AtTOC64) previously characterized in *Arabidopsis* or another plant species to bind to Hsp90/Hsp70 in our data (Table 1, Figure 2), not only validates our *in silico* search strategy but also lends support to the prediction of the newly identified CC-TPR proteins as co-chaperones of Hsp90/Hsp70. The data gathered in the present study provides many valuable insights: 1) several aspects of the Hsp90/Hsp70 TPR co-chaperone system are conserved in yeast, plant and human; 2) the Hsp90/Hsp70 TPR co-chaperone system appears to be the largest in plants due to the presence of gene families; 3) the identification of 24 new CC-TPR proteins in *Arabidopsis* with potential to act as co-chaperones indicates that the TPR domain is utilized by the Hsp90/Hsp70 machinery on a much larger scale than previously understood; 4) the presence of numerous other known functional domains in the newly identified plant CC-TPR proteins adds new functional dimensions to these proteins as well as to the Hsp90/Hsp70 chaperone machinery; 5) the plant CC-TPR co-chaperone network appears to have evolved unique features as judged by the combination of protein domains unique to plants, such as the thioredoxin domain in TTLs, and the FBD domain of unknown function in F-Box and BRCT domain containing plant proteins; and 6) the presence of a CC-TPR protein with both FBD and F-box, and another protein with protein kinase and U-box domains in rice, but not in *Arabidopsis*, indicates that some CC-TPR proteins may perform species-specific functions. Finally, the information provided here on the newly identified CC-TPR proteins will facilitate efficient investigation of their biological functions and significance. The *in silico* approach used here is the first of its kind to identify CC-TPR proteins in a genome-wide context with potential to interact with Hsp90/Hsp70 by the carboxylate clamp mechanism.

### CC-TPR proteins can bind to both Hsp90 and Hsp70

The interaction of Hsp90 and Hsp70 with TPR proteins occurs via the conserved C-terminal EEVD motif in both Hsp90 and Hsp70 and the conserved carboxylate clamp residues (K_5_N_9_-N_6_-K_2_R_6_) in the TPR domain [8]. Additional contacts involving charged and hydrophobic amino acids determine the specificity of interaction. In case of Hop, Hsp70 and Hsp90 interact with two separate domains, *viz* TPR1 and TPR2a, respectively [45], but in the case of CHIP a single TPR binds either chaperone protein [50]. This is accomplished by accommodating either the methionine of Hsp90 (DDTSR**M**EEVD) or the isoleucine of Hsp70 (GSGPT**I**EEVD) into a hydrophobic pocket of CHIP, which is not present in Hop, resulting in the peptide being twisted into a conformation so that no further specific contacts are required [50]. Additional examples of TPR proteins binding to both Hsp90 and Hsp70 include the yeast CNS1 [51], and the human Tpr2 [36]. CNS1 binds to both Hsp90 and Hsp70 with comparable affinities and while it exerts no influence on the ATPase activity of Hsp90, it activates the ATPase activity of Hsp70 up to 30-fold. Tpr2 binds Hsp90 with slightly lower affinity as compared to Hsp70, but requires ATP for binding Hsp70 in the presence of Hsp90. The DnaJ homologous J domain in Tpr2 stimulates ATP hydrolysis and polypeptide binding by Hsp70 [36]. These data bring to light the importance of TPR co-chaperones and of Hsp70 regulation in the context of the Hsp90 chaperone cycle, and raise the possibility that the TPR proteins identified herein may interact with either Hsp90 or Hsp70 or both. Experimental analyses of interaction with both Hsp90 and Hsp70 are required for each of these proteins to understand their mode of action and its implications within the context of the Hsp90/Hsp70 chaperone machinery.

### AtTPR1 and AtTPR2 bind Hsp90 through its MEEVD motif

AtTPR1 and AtTPR2 were verified for binding to Hsp90 as proof-of-concept of interaction of newly identified CC-TPR proteins with Hsp90 by the ‘carboxylate clamp’ mechanism. Both proteins bound Hsp90 in yeast two-hybrid and *in vitro* binding assays. Neither protein bound to Hsp90 lacking MEEVD, indicating that interaction occurs by the ‘carboxylate clamp’ mechanism [52], [53]. During the course of this study, a tomato TPR protein (SlTPR1) with highest similarity to AtTPR1 and Os10g34540 was demonstrated to interact with ethylene receptors, and its overexpression in tomato and *Arabidopsis* caused a range of developmental phenotypes [54]. Although no connections were made with Hsp90/Hsp70, we checked and found the presence of the consensus carboxylate clamp residues in the tomato protein, suggesting that it is an interactor of Hsp90/Hsp70. Based on the above information, it can be expected that important cellular functions will be unveiled in the future for the newly CC-TPR identified proteins.

### Putative functions of the CC-TPR proteins

The different subcellular localization possibilities and the different combinations of protein domains (Table 1, Figures 1 and 2), suggest a diverse range of functions for the CC-TPR proteins, which likely add to and/or regulate the functional capacity of the Hsp90/Hsp70 chaperone system. Proteins that have a single TPR domain and no additional known domains may function as co-chaperones by modulating the ATPase activity of Hsp90/Hsp70. It is possible that their functional specificity is derived, at least in part, through specific temporal and spatial expression patterns. Proteins with more than one TPR domain or other domains like DnaJ, ankyrin, or Phox/PBI may function additionally through recruitment of other proteins. The presence of the SET domain in AtTPR9 and Os10g36250, together with the high probability of AtTPR9 protein to be localized in the nucleus (Table 1) suggests that it cooperates with the Hsp90/Hsp70 machinery in the process of chromatin remodelling [2]. Recent identification of the targets of thioredoxins in plants suggests that these proteins could influence nearly every major cellular process [55]. The involvement of AtTTL1 in ABA signaling pathway and stress responses [35] has set the stage for further investigation of the functions of TTLs along these lines.

Subcellular localization of a protein can provide clues to its functions. AtTPR10 with one TPR domain and ankyrin repeats has a high probability of being localized to the plastid. Since the plastidial Hsp90 and Hsp70 lack the EEVD motif at their C-terminus, we speculate that the interaction of the TPR domain is limited to cytosolic Hsp90/Hsp70, which chaperones the trafficking of precursor proteins to the chloroplast [23]. Whether the ankyrin domain recruits a client for the cytosolic Hsp90/Hsp70 system or a plastidial partner for AtTPR10 itself, are questions that need to be answered in the future. Experimental documentation of the plastidial localization of AtTPR10 is a prerequisite for addressing the functions of this protein. The identification of a protein in rice (Os06g06760) that contains both protein kinase and U-box domains is intriguing. To date, no such combination of domains has been noted in a CC-TPR protein. As a unique CC-TPR protein, functional analysis of this protein should be a priority.

In conclusion, the present study has uncovered a number of new potential interactors of Hsp90/Hsp70: future investigations of these will provide a better understanding of the Hsp90/Hsp70 chaperone machinery, its functions and its mode of action in plant cells. Due to the critical requirement of the Hsp90/Hsp70 system for cell viability and normal functioning of the cell, which has ramifications in human health, knowledge of this system is a high priority in any model organism.

## Supporting Information

Figure S1 Amino acid sequence alignment of AtPhox1-4. The numbers on the side indicate the amino acid positions in the proteins. Alignment was performed using MEGA4 software. Black, grey and light grey shading indicates 100%, 75% and 50% conservation of amino acids, respectively.
(3.51 MB TIF)

Figure S2 Amino acid sequence alignment of of AtTTL1-4. The numbers on the side indicate the amino acid positions in the proteins. Alignment was performed using MEGA4 software. Black, grey and light grey shading indicates 100%, 75% and 50% conservation of amino acids, respectively.
(3.41 MB TIF)

Table S1 Sequences of primers used in quantitative RT-PCR (qPCR) analysis.
(0.03 MB DOC)
