# Supplementary figures and images for: In Silico Identification of Carboxylate Clamp Type Tetratricopeptide Repeat Proteins in Arabidopsis and Rice As Putative Co-Chaperones of Hsp90/Hsp70
Source: PLoS One. 2010 Sep 15;5(9):e12761. doi: 10.1371/journal.pone.0012761 (PMC2939883; doi:10.1371/journal.pone.0012761)

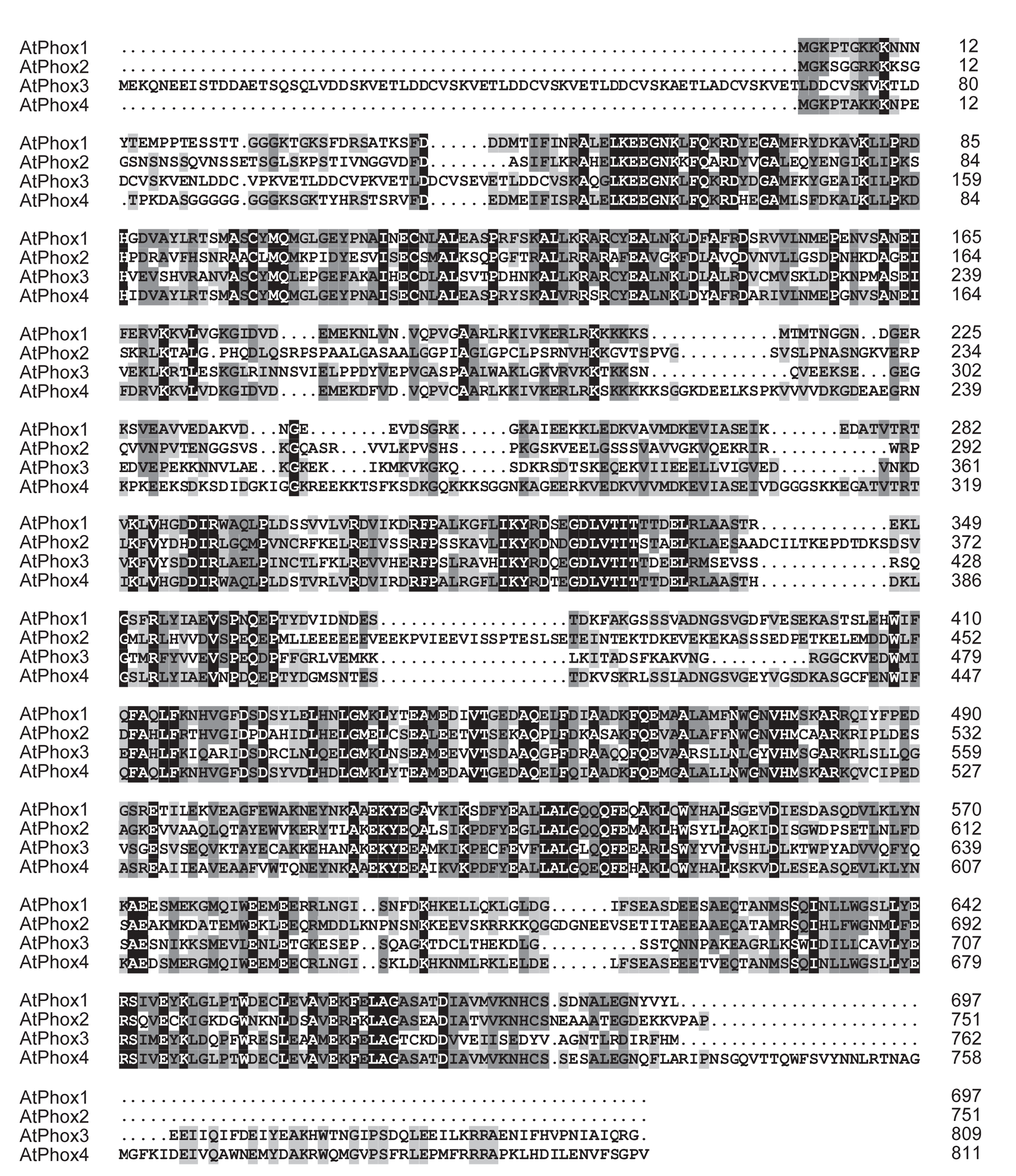

Supplement: Figure S1 — Amino acid sequence alignment of AtPhox1-4. The numbers on the side indicate the amino acid positions in the proteins. Alignment was performed using MEGA4 software. Black, grey and light grey shading indicates 100%, 75% and 50% conservation of amino acids, respectively. (3.51 MB TIF) [file pone.0012761.s001.tif]

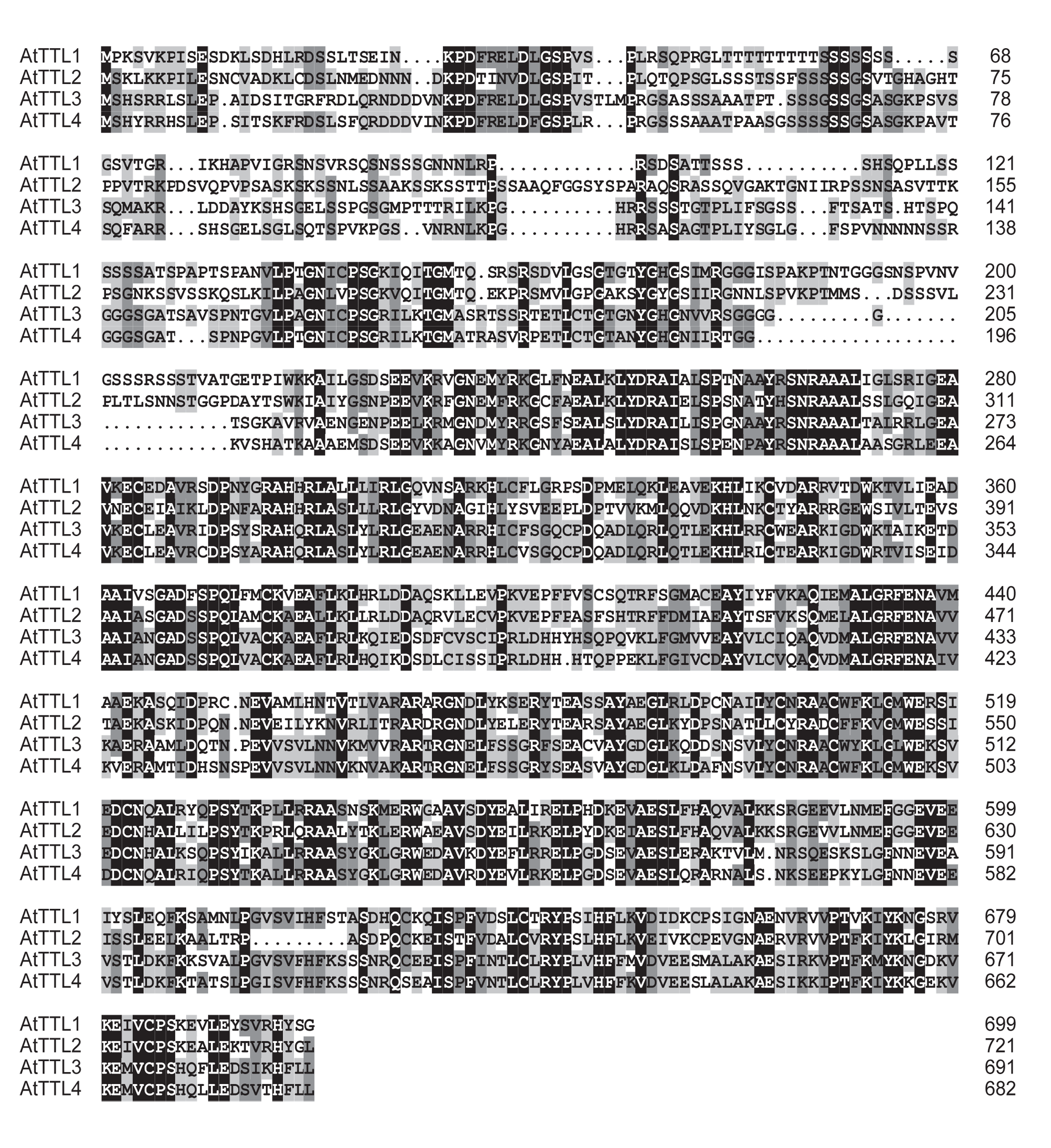

Supplement: Figure S2 — Amino acid sequence alignment of of AtTTL1-4. The numbers on the side indicate the amino acid positions in the proteins. Alignment was performed using MEGA4 software. Black, grey and light grey shading indicates 100%, 75% and 50% conservation of amino acids, respectively. (3.41 MB TIF) [file pone.0012761.s002.tif]
