# Supplementary material for: In Silico Identification of Carboxylate Clamp Type Tetratricopeptide Repeat Proteins in Arabidopsis and Rice As Putative Co-Chaperones of Hsp90/Hsp70
Source: PLoS One. 2010 Sep 15;5(9):e12761. doi: 10.1371/journal.pone.0012761 (PMC2939883; doi:10.1371/journal.pone.0012761)
Supplement: Table S1 — Sequences of primers used in quantitative RT-PCR (qPCR) analysis. (0.03 MB DOC) [file pone.0012761.s003.doc]

**Table S1.** Sequences of primers used in quantitative RT-PCR (qPCR) analysis.

| **Name** | **Forward (5’-3’)** | **Reverse (5’-3’)** |
| --- | --- | --- |
| AtTTL1 | GCTAGCCAAATCGATCCAAG | AGCTCCCCATCTTTCCATCT |
| AtTTL3 | TGTAACCATGCGCTCAAAAG | GCATTGTCGGTTTGATGATG |
| AtTTL4 | ACCCGCTGTCACTTCTCAGT | CTAACGGAGGCTCGTGTAGC |
| AtPhox2 | AGGCTCTCTCGATCAAACCA | TCATTCCCATCTCCTCCTTG |
| AtTPR2 | AACGTTAAGGCGGTGTATCG | GACAGGCCAATGAAGGATGT |
| AtTPR5 | TTGAGAACTCTTGGCGGAGT | GGGGTTGCTTTATTGCAGAA |
| AtTPR8 | TGCACTGACGGATAAGAACG | TGGCTACGTCTCTGTCGTTG |
| AtTPR10 | AAGCAAAAGCCAGAGGACCA | TGCTTTCAGGGCTAAGCAAT |
| AtTPR11 | GAAGCCAAACGCTGCTATTC | AGCACGGCGACGTAATCTAT |
| UBQ10 | CAGAACTTTGGCCGACTAC | ATGGTCTTTCCGGTGAGAG |
